# Exploring the role of ride-hailing in trip chains

**DOI:** 10.1007/s11116-022-10269-w

**Published:** 2022-03-03

**Authors:** Tanjeeb Ahmed, Michael Hyland

**Affiliations:** 1 Institute of Transportation Studies, University of California, 4000 Anteater Instruction and Research Building, Irvine, CA 92697 USA; 2 Civil and Environmental Engineering, Institute of Transportation Studies, University of California, 4000 Anteater Instruction and Research Building, Irvine, CA 92697 USA

**Keywords:** Shared mobility, Travel behavior, Activities, Ridesourcing, Trip chain, Logit choice models

## Abstract

Ride-hailing can potentially provide a variety of benefits to individuals who need to chain several activities together within a single trip chain, relative to other travel modes. Using household travel diary/survey data, the goal of this study is to assess the role ride-hailing currently plays within trip chains. Specifically, the study aims to determine, within trip chains, who uses ride-hailing services, for what trip/activity purposes, and to/from what types of areas, as well as the characteristics of trip chains that involve ride-hailing segments. To meet these objectives, the study estimates a binary logit model using 2017 National Household Travel Survey data, where the dependent variable denotes the inclusion of at least one ride-hailing trip within a trip chain. Similar to the non-trip-chaining ride-hailing literature, this study indicates that trip chains with ride-hailing legs are positively associated with travelers who are younger, live in high-income households, frequently use transit, and reside in high-density areas. However, this study includes novel findings indicating statistically significant relationships between ride-hailing and trip chains that end in healthcare and social/recreational activities. Moreover, trip chains with ride-hailing tend to have fewer stops and longer activity durations than trip chains without ride-hailing. This study also includes nested logit choice models, wherein the dependent variable denotes the primary mode (ride-hailing, transit, personal vehicle, or non-motorized transport) of a trip chain. These model results provide additional insights into the role of ride-hailing within trip chains, as they allow for cross-mode comparisons. The paper discusses the potential transportation planning and policy implications of the model results as well as future research directions.

## Introduction

A *trip chain* is a series of trips taken in which the starting and ending trips in the chain succeed and precede, respectively, a specific activity type (e.g., home and/or work) or an activity with a duration greater than some time threshold (e.g., thirty minutes or four hours). Research suggests travelers have a propensity to form trip chains to reduce travel costs and/or save time (Liu 2012; Strathman et al. 1994; Wang 2015), such as when travelers stop at non-work activity locations when traveling to or from work. In chaining trips, travelers are potentially decreasing overall vehicle miles traveled (VMT) and positively impacting the transportation system of a region (Carlson and Howard 2010; Duncan 2016).

The private automobile (i.e. auto) offers many advantages to travelers who chain their trips on a regular basis, including flexibility in scheduling and selection of routes, as well as overall travel comfort and the ability to store items between trips (Xianyu 2013). However, researchers also find that in areas with high transit demand, travelers complete complex trip chains using rail-based transit services in order to avoid roadway congestion and parking costs (Currie and Delbosc 2011).

The recent emergence and proliferation of ride-hailing companies like Uber and Lyft offers travelers another modal option to complete trip chains or portions of trip chains. In general, ride-hailing services provide many of the benefits of a private auto without the high upfront purchasing costs nor parking costs. In contrast to transit, ride-hailing services obviate the first/last mile problem by providing transportation directly from one activity location to the next. Ride-hailing trips are also a lot more flexible than transit trips in terms of scheduling and routing. Hence, ride-hailing includes most of the benefits and avoids most of the pitfalls of both personal vehicles and transit for trip chaining purposes. However, a notable downside of ride-hailing (and transit and walking) relative to a personal vehicle is the inability to store items inside a vehicle between trips. Another downside of ride-hailing is the relatively high cost per trip.

Given the potential of ride-hailing to be an attractive mode for trip chaining, the goal of this study is to explore the role ride-hailing currently plays within trip chains. The study focuses on understanding the attributes of the travelers who use ride-hailing within trip chains, the activities and trips within trip chains with ride-hailing, the complexity and structure of trip chains made with ride-hailing, and the land-use characteristics of the areas where ride-hailing trip chains typically occur. To meet the study’s overarching goal and answer specific research questions, this paper includes (i) a descriptive analysis of the characteristics of travelers across modes and across trip purposes or activities who trip chain, (ii) a binary logit model to understand the modal, individual, land-use, activity, and trip chain attributes correlated with the usage of ride-hailing within a trip chain, and (iii) a nested logit model to capture the modal, individual, land-use, activity, and trip chain attributes correlated with the choice of a primary trip chain mode. The analysis employs data from the largest, by population, 50 core-based statistical areas (CBSAs), obtained from the 2017 National Household Travel Survey (NHTS) dataset.

While several existing studies attempt to reveal trip making patterns of ride-hailing users, as far as the authors are aware, this is the first study to explore ride-hailing within the context of trip chains. Since trip chaining is a relatively complex form of travel behavior with specific travel requirements, understanding the role ride-hailing plays within trip chains should help transportation planners, policymakers, and system managers formulate policies to enable better integration of ride-hailing into people’s daily travel routines, to ultimately improve mobility and accessibility. The literature also points to the significant opportunity for ride-hailing to support multi-modal travel (Shaheen and Chan 2016), which is related to trip chaining.

The remainder of the paper is structured as follows. The next section reviews travel behavior-related ride-hailing and trip chaining research. The third section presents the theoretical framework and modeling approach to meet the study’s objectives. The fourth section provides an overview of the data used to answer the study’s research questions. The fifth section presents the model results, while the sixth section discusses the model results and their broader implications. The final section concludes the paper with a summary of the study, key findings, study limitations, and future research directions.

## Literature review

The impact of ride-hailing services on the mobility of trip makers has been quite apparent in the last few years. Their tremendous growth is evident in the nearly four-fold increase in the number of ride-hailing trips in New York City between November 2015 and November 2019 (TLC 2020). Although the demand for ride-hailing services and the supply of ride-hailing drivers diminished greatly during the COVID-19 pandemic, this study implicitly assumes that ride-hailing services will once again play an important role in urban areas in the coming years.

Previous research indicates that ride-hailing services are mostly associated with people in the middle-income group and in households with low auto access (Feigon and Murphy 2016). The same study also found a high correlation between users of ride-hailing services and transit users. In a similar study that distinguishes between young adults and middle-aged adults in California when analyzing factors impacting the use of ride-hailing services, findings indicate that ride-hailing is highly popular among younger, Hispanic, and higher-educated persons (Alemi et al. 2018). The adoption of ride-hailing was also found to be higher when individuals are more likely to associate modern technology within their daily life, make long-distance trips, and often travel to the airports. Moreover, studies indicate a significant positive influence of built environment variables, such as automobile accessibility, land use mix, and residential density on the use of ride-hailing services (Alemi et al. 2018; Dias et al. 2017). Along with relieving the traveler from the hassle of parking their car, ride-hailing services are also popular when the traveler is unable to drive and because of the convenience in access and payment (Rayle et al. 2014).

There are mixed findings regarding the impact of ride-hailing services on transit. Some studies find evidence that the use of ride-hailing services increase the use of transit depending on the location and type of transit service (Circella and Alemi 2018; Clewlow and Mishra 2017). Conversely, a study found bus ridership declined by 12.7% since 2010, when the first ride-hailing service began operation (Graehler et al. 2018). The same study also shows that between 2015 and 2018 in New York, daily ride-hailing trips increased by 540,000 whilst transit trips reduced by 580,000 (Graehler et al. 2018). While studying the change in transit ridership pertaining to 20 U.S. cities, Sadowsky and Nelson (2017) find that the introduction of ride-hailing services (Uber) had a complementary effect on the use of public transportation. But when the second company (Lyft) entered the market, they observed an opposite effect, represented by a reduction in the transit ridership to or below the level which existed before the introduction of ride-hailing services. Sadowsky and Nelson (2017) speculate that the first entry of the ride-hailing services acted as a solution to the transit first-/last-mile problem, whereas the entry of the second company created price competition between ride-hailing companies, effectively decreasing ride-hailing prices, and subsequently making ride-hailing travel a substitute for transit. Another study comprising seven metropolitan areas in the U.S. estimated that 9% of transit trips were substituted by ride-hailing services (Clewlow and Mishra 2017). Noting the potential substitution and complementary effects of ride-hailing on transit services, Circella et al. (2018) assert that the substitution effect might significantly prevail over the complementary effect, if trip makers have low or zero access to a private automobile and frequently use Uber or Lyft in combination with other modes.

Another research question pertaining to ride-hailing services is their impact on auto use and ownership since ride-hailing services provide most of the benefits of a private automobile. In San Francisco, Rayle et al. (2016) conducted intercept surveys of 380 ride-hailing users and compared the surveys with data from: the American Community Survey (ACS), a previous taxi users survey, and GPS trip records of a taxi company. Their findings indicate that 38% of ride-hailing users who own a car, drove less frequently (up to twice per week) after the introduction of ride-hailing. However, the researchers could not find any significant reduction in auto ownership among ride-hailing users. With a broader study area, covering seven metropolitan areas in the U.S., Clewlow and Mishra (2017) conducted an internet-based survey to understand the factors influencing the use of ride-hailing services. A small proportion, 9%, of the respondents reported a reduction of at least one vehicle in their households when they opted for ride-hailing services. But what these studies could not establish is whether there is any net increase in the vehicle miles traveled (VMT), which is an important metric associated with congestion, energy consumption, and vehicle emissions. Clewlow and Mishra (2017) emphasize the importance of induced VMT (i.e., by non-drivers and non-auto owners) and dead-heading VMT (i.e., VMT generated by empty ride-hailing vehicles) in their evaluation. In an effort to shed light on this issue, Henao and Marshall (2019) estimate the impact of ride-hailing on system-wide VMT through a quasi-natural experiment, where the first author drove for Uber and Lyft to obtain trip and passenger data. Results indicate that ride-hailing services increase VMT by 83.5% compared to other modes, a significant portion (40.8%) of which is attributed to the dead-heading VMT. Also, 13% of the respondents in Henao and Marshall (2019) mentioned that they are substituting ride-hailing services for auto ownership.

The findings in the prior paragraphs suggest a significant change in travel patterns and behavior due to the introduction of ride-hailing services. As established by numerous studies in the past several decades, trip chaining is an important component of travel behavior. Moreover, trip chaining has a direct effect on the way people plan their daily trips and activities. People link or chain their trips together when they have a restriction on the time and/or the day they can travel. Trip chaining can also arise simply because it is more convenient. For example, when the preferred grocery store is located along or near a traveler’s commute route, it is more efficient to add a stop when returning home from work rather than making a separate trip to the grocery store from home. Evidence suggests that travelers have a propensity to form trip chains, by adding non-work trips to work trips, with the aim to save travel cost and time (Strathman et al. 1994).

Considering trip chaining’s impact on mode choice and the spatial and temporal distribution of trips, research has attempted to utilize trip chaining characteristics to improve travel demand forecasts (Abdelghany et al. 2001; Goulias and Kitamura 1989; Krygsman et al. 2007). Trip makers were found to choose modes differently when they are making a chain of trips compared to a single direct trip. Studies also show that along with the decision to trip chain, the trip chain’s level of complexity (number of stops, cumulative activity duration, etc.) influences the primary and/or secondary modes. In highly complex trip chains, private auto is often the most preferred mode as it allows flexibility in scheduling and impromptu changes in the number and sequence of trips (Dong et al. 2006; Lee and McNally 2003). Another study by McGuckin et al. (2005), using the 2001 National Household Travel Survey dataset, reports a higher tendency to use personal vehicles when trip chains are made to and from work compared to a single trip chain in either commute direction. But there are cases, such as in a study conducted in Melbourne, where complex trip chains were highly correlated with transit (rail and tram) rather than personal cars (Currie and Delbosc 2011). Although the auto is known to provide the most flexibility, the trip chain makers in the Melbourne study choose transit for trip chaining to avoid roadway congestion and parking.

As mentioned previously, despite sizable and growing bodies of research analyzing (i) demand for ride-hailing, (ii) travel behavior related to ride-hailing in general, and (iii) trip chaining behavior in general, the authors are unaware of any other study that examines the relationship between ride-hailing and trip-chaining. Hence, this study aims to fill this gap in the literature by providing behavioral insights into the role ride-hailing currently plays within trip chains.

## Theoretical framework

Before delving into the theoretical framework underlying this study, it is important to note that this study assumes that a traveler chooses to chain a series of activities and trips prior to making mode choice decisions. While it is possible that the decision to chain trips is made simultaneously with mode choice, or that mode choice decisions are made prior to the decision to chain trips in some cases, a key study in the literature finds that the attributes of trip chains better explain mode choice, than vice versa (Ye et al. 2007).

### Operational definitions

A trip chain is usually defined by specific primary or anchor activity types (e.g., home or work), which bound the series of trips that can occur (Bautista-Hernández 2020; McGuckin and Murakami 1999). This definition, with home and work being the anchor activities, produces exactly four types of trip chains: home-to-work, work-to-home, home-to-home, and work-to-work. A different trip chain definition stems from the duration of activities, with respect to which the anchor activities are fixed. McGuckin et al. (2005) uses the Federal Highway Administration’s (FHWA’s) definition in which a trip chain terminates when an activity has a duration greater than 30 min. In this case, any activity type can serve as an anchor activity (e.g., home, work, grocery shopping, or eating out). Also, in this case, there are many different types of trip chains and the maximum number of trip chains in a day for a person is likely to be greater than the definition in which only work activities or home activities can be anchors.

The current study uses data from the 2017 NHTS (FHWA 2017) to determine the role ride-hailing currently plays within trip chains. While the NHTS dataset contains a file for trip chains, the current study does not use the NHTS trip chain dataset. The NHTS constructs its trip chain dataset in such a manner that home and work activities anchor every trip chain. Conversely, the current study constructs its trip chain dataset based on activity duration, i.e., every trip chain is anchored by activities lasting longer than four hours and the anchor points are independent of activity type. McGuckin et al. (2005) employ a similar specification for anchor points, but their anchor activities are based on a 30-min anchor activity duration cut-off. Therefore, by definition, the duration of intermediate activities within a trip chain do not exceed four hours in this study.

#### Definition 1: Trip Chain

A trip chain denotes a series of two or more trips that connect activities wherein the trip chain’s terminating activity has a duration of four hours or longer, and the intermediary activities have a duration of less than four hours.

The trip chain definition used in this study allows home and work activities to be treated as any other activity. If their durations are less than four hours, home and work activities are classified as intermediary trip activities, rather than automatically being a terminating activity for a trip chain. In the context of trip chaining, there are certainly cases in which trips ending at home locations should be considered a secondary activity, e.g., when a person needs to drop his children or perishable groceries at his home before going to another, more important (or at least time-consuming) activity.

For clarification, please note that a single trip represents travel between two activity locations. The trip can be unimodal or multimodal, where in the latter case the traveler switches modes between activity locations. Moreover, even if all the individual trips within a trip chain are unimodal, the trip chain itself can be multimodal.

**Fig. 1:**
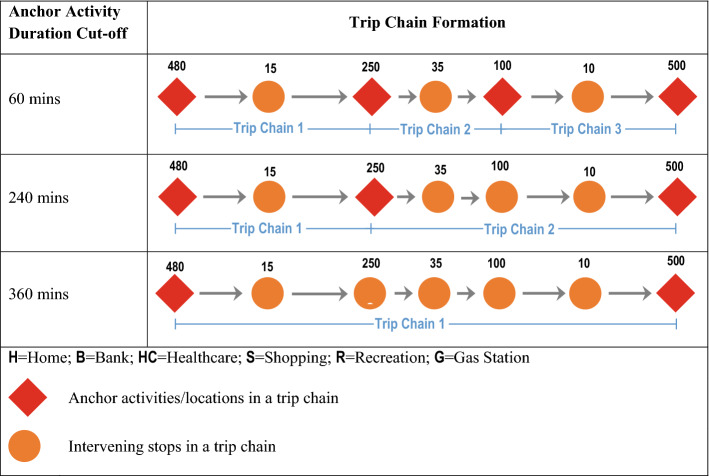
Formation of Trip Chains as a Function of Anchor Activity Duration Cut-off (values above circles represent activity durations)

Figure 1 displays how the anchor activity duration cut-off impacts the construction of trip chains. The figure shows that as the anchor activity duration cut-off increases from 60 to 240 min, to 360 min the number of trip chains decreases from three to two to one, respectively, in this example. Moreover, the average number of stops per trip chain increases from 2 to 3 to 6 in the example. In this example and all future references in this paper, the count for the number of stops in a trip chain includes the stop at the primary/terminating activity of the trip chain. Hence, according to this definition, a trip chain must have at least two stops.

**Fig. 2:**
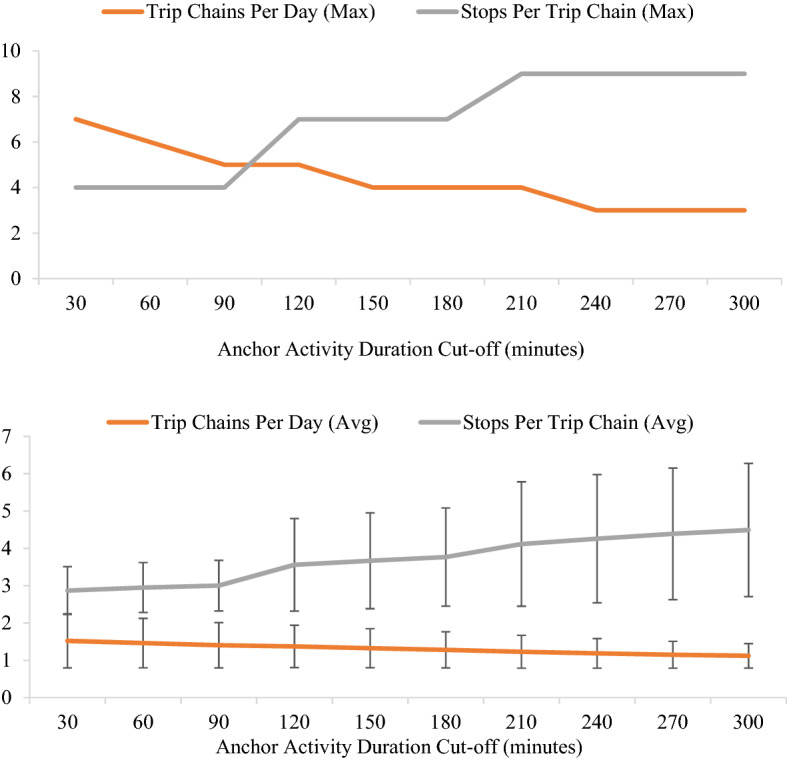
Max (Top) and Average (Bottom) Trip Chains per Day and Stops per Trip Chain as a function of Anchor Activity Duration Cut-off. (Note: Error bars represent one standard deviation)

Figure 2 displays the relationship between the anchor activity duration cut-off value and the maximum and average number of trip chains per day and the stops per trip chain. Unsurprisingly, as the anchor activity duration cut-off value increases the number of stops per trip chain increases and the number of trip chains per day decreases.

This study uses a four-hour cut-off value for anchor activity duration because lower cut-off values make it difficult to identify particularly important (or at least time-consuming) activities that a trip chain maker conducts during a day and higher cut-off values basically produce the same set of trip chains as the four-hour cut-off value. Moreover, the probability distribution of primary modes across trip chain datasets (i.e., the mode splits for the primary mode of a trip chain) changes very little when the anchor activity duration cut-off increases beyond four hours. As a result, defining trip chains with an anchor activity duration of four hours or more seems to provide a good representation of trip chains with important, or at least time-consuming, activities anchoring the trip chains.

The operational definition of trip chains resulted in 50,611 trip chains made by 42,673 persons (from 31,169 households) after filtering out missing and invalid data. The percentage of all trips in the filtered dataset that are conducted within a trip chain, as opposed to all trips, is 64%. Additionally, 52% of all persons in the filtered dataset make at least one trip chain per day. For this 52%, they average 1.2 trip chains per day per person. It should be noted that the anchor activity duration cut-off value also impacts the percentage of trips within trip chains, and the percentage of trips within trip chains increases with increases in the cut-off value.

Since a trip chain can only have one terminating anchor activity, defined as the last activity of a trip chain or the primary activity, secondary activities are those for which stops are made within a trip chain before the trip chain’s terminating stop. Therefore, a trip chain should contain at least one secondary activity, otherwise it would be a direct trip.

#### Definition 2: Primary Trip Chain Activity

The primary activity of a trip chain is the activity that terminates the trip chain because it has a duration greater than four hours.

#### Definition 3: Secondary Trip Chain Activity

A secondary activity within a trip chain is any activity that is not a primary/terminating activity within a trip chain; each secondary activity has a duration less than four hours.

#### Definition 4: Primary Trip Chain Mode

The primary trip chain mode is the mode used within the trip chain that is used to travel the farthest distance across all trips within the trip chain.

Definition 4 does not consider the number of trips made by each mode. Hence, a trip chain with five trips in which the traveler makes four of the trips via walking may have a primary mode of automobile if the distance of the single automobile trip is greater than the cumulative distance of all four walk trips.

#### Definition 5: Secondary Trip Chain Mode

A secondary trip chain mode is any mode used within the trip chain other than the primary mode; the trip chain maker travels less distance in a secondary trip chain mode than in the primary mode.

## Research questions

The goal of this study is to explore the role ride-hailing currently plays within trip chains. This goal is intentionally broad, given the exploratory nature of this research. To meet this goal and provide more specificity to guide the exploration/analysis, this subsection presents the study’s two main research questions.

1. What characteristics of trip chain makers, activities, and trips as well as the trip chains themselves and the geographical areas in which the trip chains occur, impact the propensity of trip chain makers to use ride-hailing—as a primary or secondary mode—within a trip chain?
2. What are the characteristics of trip chain modes, makers, activities, and trips, as well as the trip chains themselves and the geographical areas in which the trip chains occur that impact the likelihood a trip chain maker chooses ride-hailing as the primary trip chain mode?

The first research question parallels the second objective of the study (mentioned in the Introduction) and effectively looks to compare trip chains with one or more ride-hailing trips with trip chains with zero ride-hailing trips. The second research question parallels the third objective of the study and focuses on the primary trip chain mode and compares ride-hailing as a primary trip chain mode to auto, transit, and walking as primary trip chain modes.

## Research hypotheses

This subsection lays out the study’s hypotheses related to the two research questions posed in the previous subsection. Although different modeling approaches are needed to answer the two research questions, the hypotheses laid out in this subsection do not differentiate between the propensity of trip chain makers to use ride-hailing either as a primary or secondary mode (Research Question 1) and the likelihood a trip chain maker chooses ride-hailing as the primary trip chain mode (Research Question 2). However, this is not to say that the expectation is that the impact of each explanatory factor will be the same in the two models for the two choice situations. Conversely, because the choice situations are fundamentally different—Research Question 1 and the associated model focus on the choice of ride-hailing as either a primary or secondary mode within a trip chain, while Research Question 2 and the associated model focus on the choice of ride-hailing as the primary mode only—the strong expectation is that the factors, particularly their relative magnitudes, will vary significantly across the two models. However, given the exploratory nature of the research study, and the dearth of existing theoretical and empirical research on ride-hailing in trip chains, the authors do not have specific a priori expectations for the relative differences between most of the factors in each of the two models. One notable exception is for the stops per trip chain variable, where the expectation is that ride-hailing usage will decrease with stops per trip chain in both choice contexts but that the magnitude will be significantly higher for ride-hailing as the primary trip chain mode choice context. The reason being that while ride-hailing may support complicated multi-modal trip chaining, the authors do not expect travelers to make complicated, many stop, trip chains exclusively or predominantly using ride-hailing given the costs of ride-hailing and the inability to store items between trips.

This subsection covers hypotheses related to modal attributes, trip chain maker characteristics, activity types, trip chain complexity and structure, and land-use characteristics. At a high level, this study hypothesizes that each of these categories of factors/variables will have a causal impact on the use of ride-hailing within trip chains, and trip chain mode choice in general.

Regarding modal attributes, given data limitations, this study does not focus on modal attributes. However, the statistical models do incorporate cumulative trip chain travel time and cumulative trip chain wait time, where the latter is only associated with transit trips. Naturally, the expectation is that as travel time and wait time increase, a mode becomes less attractive as a primary trip chain mode. Even though modal attributes are critical to explaining variance in mode choice, this study focuses on the other attributes that also have important implications for forecasting travel behavior and planning transportation systems. Other than potentially land-use attributes and the trip chain structure and complexity variables, the exclusion of other modal attributes from the statistical models is unlikely to bias the non-modal parameter estimates, as these factors are generally not systematically correlated with modal attributes.

Trip chain maker attributes includes socio-demographic characteristics as well as transportation and travel related characteristics. Based on the empirical literature related to mode choice for individual trips, the expectation is that young, high-income, white males with a secondary degree and a full-time job are the most likely to use ride-hailing within trip chains (Dias et al. 2017). Hence, the expectation is that older persons, persons from lower income households, non-white persons, females, persons with lower education attainment levels, and unemployed and part-time workers are less likely to use ride-hailing within trip chains.

Regarding transportation characteristics related to trip chain makers, the expectation is that persons who live in households with ample vehicles and persons who do not tend to use transit are less likely to use ride-hailing within trip chains. The vehicle availability hypothesis stems from the idea that people who have ample access to a private auto would not benefit much from the attributes of ride-hailing, compared to walking and transit, in terms of trip chaining because one’s own auto is usually superior (except when parking costs are quite high). Moreover, empirical evidence suggests people with insufficient vehicles have a higher likelihood to use ride-hailing (Sikder 2019). The transit usage hypothesis stems from empirical research in the literature finding that transit users tend to be likely users of ride-hailing (Feigon and Murphy 2016).

Regarding primary trip chain activities, although the expectation is that primary activities do impact the use of ride-hailing with trip chains, the magnitude and directionality for individual activity types is mostly uncertain. One notable exception is the eating out or meal activity, in which prior research suggests that areas within cities that have more restaurants tend to have higher usage of ride-hailing services (Ghaffar et al. 2020).

Another set of attributes of interest relate to the structure and complexity of the trip chain as a whole. The variables included in the statistical models related to trip chain complexity include cumulative activity duration, cumulative travel distance or cumulative travel time, and total number of stops. The expectation is that all three attributes are likely to impact the likelihood of ride-hailing existing within a trip chain. However, there is no a priori definitive reasoning for the directionality of the impacts of these three parameters. Compared to private auto, ride-hailing trip chains are probably likely to have fewer stops, given the ability to park a personal auto and store personal items between trips. However, compared to transit, ride-hailing trip chains may have more stops, given the added convenience of door-to-door service provided by ride-hailing.

The trip chain anchor activities variable included in the statistical models captures both the activity location/type at the beginning and the end of the trip chain. Similar to the primary trip chain activity, which only considers the activity location/type that terminates the trip chain, the trip chain anchor activities variable is likely to impact the use of ride-hailing within trip chains, but the directionality of the relationship is mostly unclear a priori, except for the home-based socio-recreational anchor activity pairing.

Finally, another expectation is that land-use, specifically density, will impact the use of ride-hailing within trip chains. Given previous research using travel survey data (Dias et al. 2017), as well as previous research using ride-hailing count data and models (Ghaffar et al. 2020), there is a clear expectation that higher density regions are likely to have higher usage of ride-hailing within trip chains.

### Modeling approach

To answer the questions proposed in the Research Questions subsection and to test the hypotheses laid out in the Research Hypotheses subsection, this study employs two different statistical modeling techniques. The first, focused on the first research question, is the binomial logit (BL) model. The second, focused on the second research question, is the nested logit (NL) model. These two models are laid out in the following two subsections. For a more detailed overview of the derivation of these models, please refer to Train (2009) and Ortúzar and Willumsen (2011). In fact, much of the descriptions below are paraphrased from these two sources.

#### Binomial logit

The first research question is interested in the propensity of a trip chain maker to incorporate ride-hailing as a primary or secondary trip chain mode, as a function of several categories of variables:

$U$: vector of attributes associated with the trip chain maker.

$C$: vector of attributes associated with trip chain structure, complexity, and activities.

$L$: vector of land-use attributes associated with the city/region where the trip chain occurs.

Moreover, let ${X}_{i}$ denote the set of all relevant attributes associated with trip chain $i$; ${X}_{i}=\left\{U,C,L\right\}$.

To model the propensity of a trip chain maker to use ride-hailing in a trip chain with attributes ${X}_{i}$, this study employs the latent variable representation of the binomial logit (BL) model. Letting ${y}_{i}^{*}$ represent the unobserved or latent propensity of the trip chain maker to use ride-hailing in trip chain $i$, the following mathematical relationships in Eq. 1–2 describe the latent variable representation of the BL model.

1 $$y_{i}^{*} = \beta X_{i} + \varepsilon_{i}$$

2 $$y_{i} = \left\{ {\begin{array}{*{20}c} 1 & {y_{i}^{*} \ge 0} \\ 0 & {y_{i}^{*} < 0} \\ \end{array} } \right.$$

In Eq. 2, ${y}_{i}$ is a binary observable variable equal to one if trip chain $i$ includes ride-hailing and zero otherwise. In Eq. 1, $\beta$ is a vector of parameters/coefficients associated with the variables in ${X}_{i}$. Moreover, ${\varepsilon }_{i}$ represents the unobservable attributes impacting the trip chain maker’s propensity to include ride-hailing within trip chain $i$. Under the assumption that ${\varepsilon }_{i}$ follows the standard logistics distribution, the probability that trip chain $i$ includes ride-hailing (${P}_{i}$) is shown in Eq. 3, where $\alpha$ is a constant parameter associated with ride-hailing’s inclusion in the trip chain.

3 $$P_{i} = \frac{{\exp \left( {\alpha + \beta X_{i}^{^{\prime}} } \right)}}{{1 + \exp \left( {\alpha + \beta X_{i}^{^{\prime}} } \right)}}$$

To estimate the parameters $\alpha$, $\beta$ in Eq. 3, standard maximum likelihood estimation techniques can be used. These techniques are employed in STATA, the statistical modeling software used in this study.

#### Nested logit

The second research question is interested in the propensity that a trip chain maker chooses ride-hailing or another mode $m$ as their primary trip chain mode in trip chain $i$. To address this research question, this study proposes a utility maximization framework (Eq. 4), i.e., a trip chain maker will choose the mode $m$ with the highest utility for trip chain $i$.

4 $$m = \arg \max \left( {U_{i,m} } \right)$$

In Eq. 4, ${U}_{i,m}$ is the utility of mode $m$ for trip chain $i$. However, because utility is not fully observable, it needs to be separated into the observable component ${V}_{i,m}$ and unobserved component ${\varepsilon }_{i,m}$; ${U}_{i,m}={V}_{i,m}+{\varepsilon }_{i,m}$. The observable component is the product of explanatory variables ${X}_{i,m}$, which are the same as in the previous subsection with the addition of attributes associated with mode $m$, and the vector of associated parameter values estimated, ${\beta }_{m}$, of which some depend on the mode $m$. Assuming the unobserved component ${\varepsilon }_{i,m}$ is independently and identically disturbed across modes and trip chains and it follows the Gumbel distribution, then the probability that the trip chain maker chooses mode $m$ for trip chain $i$ is shown in Eq. 5.

5 $$P_{{i,m}} = \frac{{\exp \left( {\alpha _{m} + X_{{i,m}} \beta _{m} } \right)}}{{\sum\nolimits_{{k = 1}}^{{\left| M \right|}} {\exp } \left( {\alpha _{k} + X_{{i,k}} \beta _{m} } \right)}},\;\forall m \in M$$

where$${P}_{i,m}$$: probability that individual $$i$$ chooses mode $$m$$$$;\, {\alpha }_{m}$$: mode-specific constant for mode $$m$$$$;\,{X}_{i,m}$$: vector of attributes associated with mode $$m$$ for trip chain $$i$$$$;\,{\beta }_{m}$$: vector of coefficients for mode $$m$$$$;\,M$$: the set of modes $$M$$, indexed by $$m\in M$$, which includes auto, non-motorized transport or NMT (walk, bicycle), ride-hailing, and transit (bus, rail).

Equation 5 displays the multinomial logit (MNL) model with the independence of irrelevant alternatives (IIA) property that implies the error terms across all modes are assumed independent or uncorrelated. However, due to data limitations it is often the case that the error terms are correlated across modes, and different error term assumptions are necessary. This study employs the nested logit (NL) model that groups discrete alternatives into nests, in which the NL model captures correlation across modes within a particular nest, meaning that IIA holds within nests but not across nests. The Specification and Estimation of the NL and MNL Model subsection provides details on the different nesting structures tested and the selected nesting structure for this study.

Let $N$ denote the set of nests, indexed by $n\in N$. Also, let ${B}_{n}$ denote the set of mode alternatives in nest $n$ and let ${n}_{m}$ denote the nest of mode $m$. The NL model assumes that the vector of unobserved utility components ${\varepsilon }_{i}=\left[{\varepsilon }_{i,1}, \dots ,{\varepsilon }_{i,\left|M\right|}\right]$ has the following cumulative distribution in Eq. 6.

6 $$\exp \left( { - \mathop \sum \limits_{n \in N} \left( {\mathop \sum \limits_{{j \in B_{n} }} \left( {e^{{ - \frac{{\varepsilon_{i,j} }}{{\lambda_{n} }}}} } \right)} \right)^{{\lambda_{n} }} } \right)$$

The marginal distribution of each ${\varepsilon }_{i,j}$ is univariate Gumbel but the ${\varepsilon }_{i,j}$’s are correlated within nests—they are not correlated across nests. Moreover, the parameter ${\lambda }_{n}$ measures the degree of independence in unobserved utility among the alternatives in nest $n$. Or put alternatively, $1-{\lambda }_{n}$ is a measure of correlation for the alternatives in a nest $n$. Hence, if ${\lambda }_{n}=1$, the alternatives in a nest are uncorrelated and the NL model reduces to the MNL model.

Given the cumulative distribution in Eq. 6, the probability that a trip chain maker chooses mode $m$ for trip chain $i$ is displayed in Eq. 7.

7 $$P_{{i,m}} = \frac{{\exp \left( {\frac{{V_{{i,m}} }}{{\lambda _{{n_{m} }} }}} \right)\left[ {\sum\nolimits_{{j \in B_{{n_{m} }} }} {\exp } \left( {\frac{{V_{{i,j}} }}{{\lambda _{{n_{m} }} }}} \right)} \right]^{{\lambda _{{n_{m} }} - 1}} }}{{\sum\nolimits_{{q = 1}}^{N} {\left[ {\sum\nolimits_{{k \in B_{q} }} {\exp } \left( {\frac{{V_{{i,k}} }}{{\lambda _{q} }}} \right)} \right]^{{\lambda _{q} }} } }},\;\forall m \in M$$

where $${P}_{i,m}$$: probability that the trip chain maker chooses mode $$m$$ for trip chain $$i$$$$;\,{V}_{i,m}$$: deterministic component of the utility for mode $$m$$ in trip chain $$i$$, where: $${V}_{i,m}={\alpha }_{m}+{X}_{i,m}{\beta }_{m}$$. The terms have the same meaning as in the MNL model$$;\,{{\lambda }_{n}}_{m}$$: logsum parameter of mode $$m$$’s nest $${n}_{m}$$ denoting the degree of independence in unobserved utility among the alternatives in nest $${n}_{m}.$$

Similar to the BL model, the NL model and the MNL model can be estimated using standard maximum likelihood estimation techniques and such as those built into STATA, the statistical modeling software used in this study.

## Data overview

### Data source

This study relies on the household, person, and trip-level information from the 2017 NHTS, which is one of the few publicly available datasets that includes ride-hailing data. Based on the population density, this study analyses the 50 largest core-based statistical areas (CBSAs) from this dataset. Along with detailed information on daily trip, person, and household characteristics, the NHTS dataset also includes demographic information, such as the population and residential density.

Using the aforementioned data and definition of a trip chain (discussed in the Operational Definitions subsection), daily trips of each person were grouped into trip chains. The resulting dataset, where an observation is a trip chain, was then analyzed with the help of descriptive statistics to have a preliminary understanding of trip chaining, associated mode choice, and the primary and secondary activities within each trip chain.

The dataset contains a mode choice indicator for each trip. All persons in the dataset chose one or more of nine mode options to complete trip chains. To have a more manageable statistical model, the observations were further restricted to represent a choice set of four primary transportation modes, namely, auto (all private vehicles including SUV and pickup trucks), non-motorized transport or NMT (walk, bicycle), ride-hailing, and transit (bus, rail). The excluded mode options (motorcycle, rental, and other modes) were not considered important in the context of this study due to their low share.

In the 2017 NHTS, the mode category for ride-hailing includes both taxis and trips ordered through transportation network companies (TNCs). Since this study defines ride-hailing as those trips provided by app-based TNC services, the study attempts to separate TNC trips from taxi trips, using a person-level variable that provides frequency of TNC app usage in the past month. This study categorizes trips made by persons who reported at least one TNC app usage in the past month as ride-hailing trips. Conversely, trips made by persons who reported zero TNC app usage are considered as taxi trips. Assuming accurate reporting by respondents, this categorization effectively removes trips from the ride-hailing category by travelers who definitely did not make a TNC trip. Conversely, a traveler who reports having used a TNC app in the past month may also have made taxi trips. However, the existing data does not allow us to fully distinguish between taxi and TNC trips. Nevertheless, the proposed classification should allow modelers and analysts to obtain a reasonable understanding of ride-hailing’s role within trip chains. The final model does not include taxi as a fifth primary mode because taxi is the primary trip chain mode in only 0.1% of trip chains, which resulted in non-convergent models.

### Filtering

Before finalizing the trip chain dataset for analysis, the dataset was filtered. First, the dataset was filtered to ensure the trips and trip chains in the data were not based on highly irregular travel and activity patterns for an individual. Hence, entire person-days of travel were removed from the dataset if (i) they did not start their first trip of the day from home, or (ii) they did not return home at the completion of their last trip of the day.

Second, trips were filtered based on trip purpose in the NHTS dataset. Unfortunately, the publicly available 2017 NHTS dataset does not provide a separate activity dataset, so this study treats the trip purpose field provided in the trip dataset as the main activity at the end of each trip. For filtering, trips that were made for the purpose of exercise (e.g., jogging) were removed as they do not have an activity at the end of the trip. Additionally, trips that were made for the purpose of mode changes (e.g., walk trip to transit station) were combined with the next trip leg to form a multi-modal trip leg in an activity-trip chain.

Third, distances and speeds were checked for each mode used in trips and all seemingly invalid observations were removed. Specifically, persons reporting distances greater than 3, 10, 80, 80, 40 and 40 miles when traveling with walk, bike, auto, transit, ride-hailing, and taxi, respectively, were removed from the dataset. In addition, those persons were removed from the dataset whose trips resulted in a calculated average speed greater than 5, 15, 70, 70, 70 and 70 miles per hour for walk, bike, auto, transit, ride-hailing, and taxi, respectively. Similarly, persons with travel speeds lower than 0.5, 2, 3, 3, 3 and 3 miles per hour for the aforementioned modes were also removed.

Fourth, trip chains starting and ending at the home location with one intervening stop were removed from the dataset. This sequence of activities and trips is a simple round-trip and does not reflect a true trip chain.

Lastly, outliers at the trip chain level (e.g., total travel time, total travel distance, and total number of stops) were also removed. Specifically, all trip chains with total travel time less than 5 min or greater than 720 min were filtered from the dataset. In the case of total travel distance, the lower bound was set at 0.1 miles while the upper bound was unrestricted as unreasonably long travel distances were captured through the other trip and trip chain filters. The maximum value of the total number of stops in a trip chain was based on the following formula: 1.5 times the interquartile range above the third quartile of the distribution. According to this calculation, the maximum allowable value for the number of stops is 9.5.

After filtering, the dataset contains observations pertaining to 42,673 persons from 31,169 households. The original dataset contained 97,453 persons and 50,982 households for the selected study area.

Before specifying the variables in the logit models, a collinearity check was conducted using variance inflation factor (VIF) and pairwise Pearson correlations. Considering all the regressors in the models, the maximum VIF was 3.47 with a minimum tolerance of 0.30, which is well within the acceptable ranges (Pearson 2012). The correlation matrix of the model regressors presented in Table 5 of the Appendix also indicates that the magnitude of any of the pairwise correlations does not exceed 0.50 in most of the cases, while none of them exceed 0.80.

### Descriptive analysis

Table 1 displays a preliminary descriptive analysis of trip chain patterns. The results indicate that the median (average) number of stops is four (four, respectively) for residents of the study area and the median (average) stop length is 112 (152) minutes. The median trip chain distance and trip chain travel duration are 22 miles and 70 min, respectively. Although the number of stops in a chain seems to vary widely, around 80% of the trip chains are limited to five stops or less.

**Table 1:** Trip chain statistics

Statistics	Number of Stops	Total Activity Duration (mins)	Total Travel Distance (miles)	Total Travel Time (mins)
Min	2	1.0	0.11	5.0
Max	9	913	494	697
Average	4.3	152	30.2	83
Median	4	112	22.2	70
Standard Deviation	1.7	142	28.6	56

As anticipated, the proportion of trip chain makers using ride-hailing as the primary mode is very low (0.28%) compared to other modes. The primary mode share for non-motorized transport (NMT), auto, transit, and ride-hailing are 3.9%, 93.3% 2.5%, and 0.3%, respectively. Clearly, as expected, the automobile is the most preferred mode among trip chain makers.

**Fig. 3:**
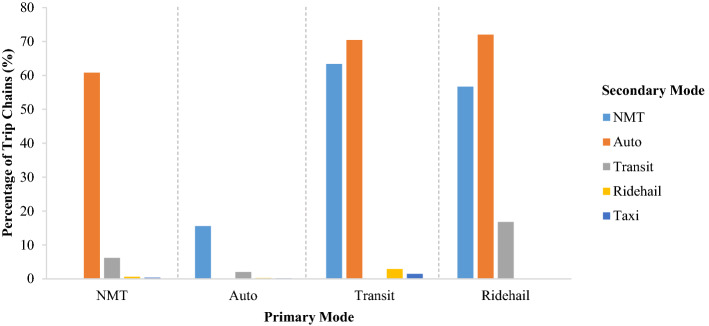
Distribution of Secondary Modes with respect to Primary Modes

Figure 3 displays the distribution of secondary modes in a trip chain conditional on the primary mode of the trip chain. The height of the bar indicates the proportion of all the trip chains with a specific primary mode containing at least one trip with the secondary mode. Notably the secondary mode shares do not have to sum to 100%. In the case of NMT primary mode, 61% of these trip chains also include auto, 6% transit, and less than 1% for ride-hailing and taxi. This indicates that many NMT trip chains do not include a secondary mode. Conversely, in the case of ride-hailing primary mode, 57% and 72% of these trip chains also include NMT and auto, respectively, and 17% include transit. This indicates that a large percentage of ride-hailing trip chains do have at least one secondary mode if not two.

The results in Fig. 3 indicate that NMT and auto are quite popular secondary modes. The share of NMT as a secondary mode is particularly high for transit and ride-hailing. The share of auto as a secondary mode is particularly high for transit and ride-hailing but is also dominant in NMT trip chains. Interestingly, auto is more frequently the second mode of transit trip chains than NMT. An investigation into the trip chain dataset reveals that auto trip segments are common both before and after the transit leg in a trip chain. Regarding the presence of auto trip after transit, the dataset provides evidence of mode switching occurring at home, which is possible due to the specific definition of trip chain used in this study. It is also possible that the person using transit is picked up from the transit station by another person who is using auto.

The percentage of ride-hailing trip chains where NMT is present is quite high and comparable with transit trip chains. In contrast, the proportion of NMT is lowest (compared to its proportion in other primary modes) when auto is the primary mode. This behavior is likely because trip makers have less incentive to use other modes when they are already using auto within a trip chain.

Trip chains with ride-hailing as the primary mode are multi-modal in many cases (over 96%) and include NMT, automobile, and transit as secondary modes. On the other hand, ride-hailing is rarely used as a secondary trip chain mode, which is not surprising given its low overall share. Although the share is very low (3%), the highest presence of ride-hailing as a secondary mode is found in transit trip chains where the presence of taxi is also evident (1%).

**Fig. 4:**
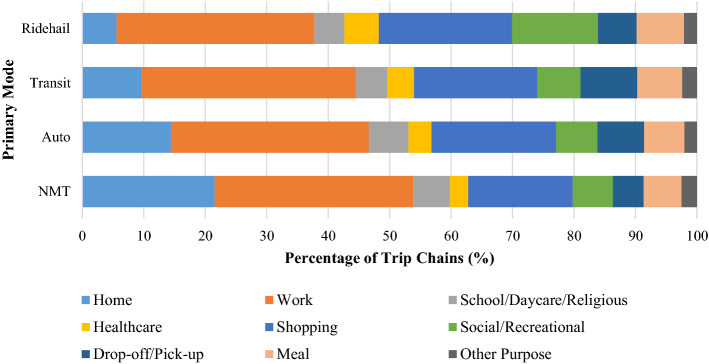
Distribution of Primary Trip Chain Activities across Primary Modes

Figure 4 illustrates the distribution of primary trip chain activities across the four primary mode categories. Overall, work and shopping have a considerable share in all primary travel modes, and they also have the highest shares among all primary activities (33.3% and 21.7% trip chains respectively). Home is highly dominant in NMT trips followed by work and shopping. The distribution of primary trip chain activities is similar between auto and transit, except the latter has a slightly higher share of work trips.

While travelers mostly choose ride-hailing in trip chains when they are traveling for work and shopping activities (53.8%), compared to other modes, ride-hailing has the highest share of trip chains pertaining to healthcare, shopping, social/recreational, and meals. Ride-hailing is used less frequently for home and drop-off/pick-up trips than the other primary modes. This is an interesting finding and suggests that people are using ride-hailing services for significantly (in a practical sense) different primary trip chain activities than existing travel modes. The role of ride-hailing in terms of transporting people to healthcare-based activities is likely something that researchers, transportation analysts, and policymakers should continue to monitor and consider in planning and policy making. The Choice Model Results section provides more detailed insights into this relationship, after controlling for other potentially spurious factors.

**Fig. 5:**
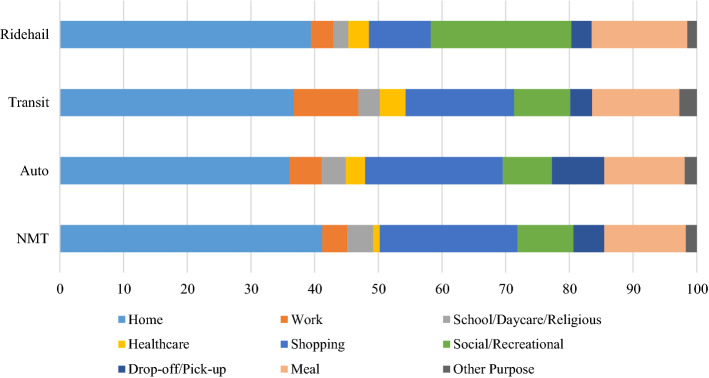
Distribution of Secondary Trip Chain Activities across Primary Modes

Figure 5 shows the distribution of secondary trip chain activities across primary modes. Home, shopping, and eating-out are dominant across all modes. Social/recreational and eating-out appear to have the highest share of trip chains where the primary mode is ride-hailing; shopping and drop-off/pickup have the highest share where the primary mode is auto; whereas work and healthcare related stops have the highest share when the primary mode is transit.

Table 2 depicts the distribution of selected variables for the mode choice model across the four primary trip chain mode alternatives. The table includes socio-demographic characteristics of trip chain makers, travel characteristics of trip chain makers, trip chain structure and complexity, activity characteristics, and land-use characteristics. Only average transit wait time is excluded as it is only relevant to the transit mode. Except gender, the cross-mode distributions of all the variables are found to be significantly different considering the *p*-value of the chi-square test or ANOVA test.

**Table 2:** Descriptive statistics for four trip chain modes

Variables	NMT (N = 1,992)	Auto (N = 47,229)	Ride-hailing (N = 143)	Transit (N = 1,252)
Socio-demographics of trip chain maker				
Gender				
Male	48.04	45.78	46.85	47.76
Female	51.96	54.22	53.15	52.24
Age (years)				
16–35	31.43	20.51	55.94	34.19
36–65	48.29	54.39	32.87	51.52
66 +	20.28	25.10	11.19	14.30
Household income (USD)				
Low (≤ 25,000)	23.64	9.21	14.69	24.04
Lower middle (25,000 to < 50,000)	15.96	15.95	6.99	14.06
Middle (50,000 to < 100,000)	24.05	31.59	27.27	23.72
Upper middle (100,000 to < 200,000)	24.10	32.19	27.97	25.32
High (≥ 200,000)	12.25	11.05	23.08	12.86
Ethnicity/race				
White	67.04	74.11	72.03	56.38
Black	8.05	6.39	4.09	16.76
Asian	10.62	6.67	11.89	10.02
Hispanic	10.42	9.85	6.99	12.75
Other race	3.88	2.99	4.20	4.09
Education				
Below bachelor’s degree	42.48	43.90	20.98	36.21
Bachelor’s degree and above	57.52	56.10	79.02	63.79
Life cycle status				
Working adult without children	42.97	34.48	72.73	58.15
Working adult with children 0–15	24.60	26.37	8.39	17.17
Working adult with children 16–21	5.12	7.32	4.20	6.47
Retired adult without children	27.31	31.84	14.69	18.21
Employment status				
Unemployed	50.80	40.86	30.50	32.11
Part-time	12.52	11.97	8.51	11.57
Full-time	36.68	47.17	60.99	56.32
Travel characteristics of trip chain maker				
Public transit usage (days per month)	4.30	0.61	8.04	17.87
Vehicle availability				
Low (< 1 vehicle per driver)	39.91	9.80	44.76	55.27
High (1 + vehicle per driver)	60.09	90.20	55.24	44.73
Day of week				
Travel day				
Weekend	26.41	22.09	32.87	11.90
Weekday	73.59	77.91	67.13	88.10
Trip chain complexity and structure				
Cumulative activity duration (mins)	111.11	153.15	202.71	179.65
Cumulative travel time (mins)	59.71	82.26	85.08	141.56
Stops per trip chain	3.53	4.29	4.00	4.17
Trip chain activities				
Trip chain anchor activities				
HBW	8.89	21.98	29.37	38.50
HBSHOP	3.97	3.08	2.10	1.04
HBSOCREC	1.61	1.89	14.69	1.60
HBO	12.10	11.07	9.79	9.66
NHB	73.44	61.97	44.06	49.20
Primary trip chain activity (main purpose)				
Home	21.44	14.44	5.59	9.66
Work	32.38	32.19	32.17	34.90
School/daycare/religious	5.97	6.42	4.90	5.11
Healthcare	2.96	3.74	5.59	4.31
Shopping	17.02	20.26	21.68	20.05
Social/recreational	6.53	6.74	13.99	7.03
Drop-off/pickup	5.02	7.59	6.29	9.27
Meal	6.17	6.59	7.69	7.27
Other	2.51	2.04	2.10	2.40
Land-use				
Residential density (HU per sq. mile)				
Low (0–499)	7.73	24.87	2.10	5.43
Medium (500–1,999)	23.24	42.89	14.69	19.57
High (2,000–9,999)	46.39	30.43	38.46	42.65
Very high (10,000–999,999)	22.64	1.81	44.76	32.35
Average household vehicle ownership in CBSA	1.76	1.84	1.75	1.65

Note 1: Means are only used for continuous variables. For categorical variables, percentage distribution is used

Note 2: For each variable, except gender, there is a statistically significant difference across the four modes

Looking at the socio-demographic variables, there is almost no variation in gender across the use of primary modes. As expected, automobile and ride-hailing trip chains are associated with medium to high income groups. Although ride-hailing is typically cheaper than a conventional taxi, the cost is still high and comparable to private car, which might explain its use by higher income trip makers. Buehler and Hamre (2015) find that high income groups have a greater tendency to make multi-modal trips than other income groups. Another significant difference across modes is found in the distribution of life cycle status. The majority (87.4%) of trip chain makers using ride-hailing do not have children in their household and a large proportion of them are employed. As travelling with children sometimes requires setting up car seats, it is unlikely that parents would opt for ride-hailing or transit when making multiple trips, especially if they own a vehicle. Finally, the distribution of age and education across modes, particularly ride-hailing, are consistent with much of the existing literature.

The variation across the modes in terms of travel day of the week is also quite high. The data shows that 33% of ride-hailing trip chains are made on the weekend compared to 12% of transit, 22% of auto, and 26% of NMT.

Considering the variables pertaining to travel characteristics, there is considerable variation in public transit usage and vehicles per driver across the four modes. Apart from transit trip chain makers, ride-hailing users have the second highest rate of public transit usage. Also, most of the trip chain makers across NMT, automobile, and ride-hailing have at least one vehicle per driver in their household. This suggests that trip chains with NMT and ride-hailing as primary modes are possibly substituting non-auto modes for auto trips. However, it is important to note that vehicle availability is the highest for auto (90.2%) trip chains followed by NMT (60.1%), then ride-hailing (55.2%) and transit (44.7%).

There is also considerable variation across the four modes in terms of activity duration, travel time duration, and stops per trip chain. Cumulative activity duration and cumulative travel time denote the total time within a trip chain conducting activities and traveling between activities, respectively. In the NL model, the cumulative travel time varies by primary mode in the choice model. Given trip chain $i$, the cumulative travel time for mode $m$ is calculated as shown in Eq. 8,

8 $$CTT_{i,m} = \mathop \sum \limits_{{t \in Trips_{i} }} 1_{{m_{t} = m}} \times tt_{t} + \mathop \sum \limits_{{t \in Trips_{i} }} 1_{{m_{t} \ne m}} \times \frac{{d_{t} }}{{u_{m} }}$$

where $$CT{T}_{i,m}$$: cumulative travel time for mode $$m$$ in trip chain $$i$$$$;\,Trip{s}_{i}$$ : set of trips in trip chain $$i$$, indexed by trip $$t\in Trip{s}_{i}$$$$;\,{1}_{A}$$ : indicator function that returns a value of 1 if $$A$$ is true, and zero otherwise$$; {m}_{t}$$ : travel mode of trip $$t$$$$;\,t{t}_{t}$$: travel time of trip $$t$$$$;\,{d}_{t}$$: distance of trip $$t$$$$;\,{u}_{m}$$: average speed of mode $$m$$, where $${u}_{auto},{u}_{NMT},{u}_{transit}, {u}_{RH}$$ are 22.3, 4.2, 11.9, and 13.8 mph, respectively.

For example, if a traveler originally made three trips with distances 2 miles, 10 miles, and 15 miles via NMT, auto, and ride-hail, respectively, then Table 3 below shows the actual travel times for each leg of the trip as well as how the cumulative travel time is computed for each trip chain mode $m\in M$ as in Eq. 8. For the ride-hailing mode option in the last row, Trip 3 was originally made with ride-hailing so the first term on the right-side of Eq. 8 is active and 55 min is the value used in this cell. Conversely, because Trip 1 and Trip 2 were not made with ride-hail, the second term on the right-side of Eq. 8 is active and used to compute/estimate the travel time in the counterfactual scenario where ride-hailing was used to conduct these two trips.

**Table 3:** Example calculation of cumulative trip chain travel time by mode, based on Eq. 8

Mode	Trip			Cumulative Trip Chain Time
	Trip 1 Distance: 2 miles Actual Mode: NMT	Trip 2 Distance: 10 miles Actual Mode: auto	Trip 3 Distance: 15 miles Actual Mode: ride-hail	
Actual	33 min	22 min	55 min	110 min
Auto	2mi/22.3mph = 5.4 min	**22 min**	40.4 min	67.7 min
NMT	**33 min**	142.9 min	214.3 min	390.1 min
Transit	2mi/11.9mph = 10.1 min	50.4 min	75.6 min	136.1 min
Ride-hail	2mi/13.8mph = 8.7 min	43.5 min	**55 min**	107.2 min

The BL models includes cumulative travel distance, which is the same independent of mode so is not displayed in Table 2 in place of cumulative travel time. The BL model does not include cumulative travel time because cumulative distance effectively captures the choice context in which the trip chain maker finds themself, when considering whether to use ride-hailing for one or more of the trip chain segments.

Ride-hailing trip chains have the highest activity durations followed by transit, auto, and NMT, with ride-hailing trip chain activity durations being more than 50 min longer than auto. Regarding, trip chain travel time, transit trip chains easily have the longest travel time durations at 142 min, followed by ride-hailing and auto between 80 and 85 min, and then NMT at 59 min.

The trip chain anchor activities variable captures the activities at the beginning and end of a trip chain. The study includes five different anchor activity combinations, namely home-based work (HBW), home-based shopping (HBSHOP), home-based social/recreation (HBSOCREC), home-based other (HBO), and non-home based (NHB). Trips where one start or end activity type/location is shopping, and the other activity type/location is home are labeled HBSHOP. The HBO option captures the case where one start or end activity type/location is home and the other activity type/location is not working, social/recreation, or shopping. Finally, NHB denotes the case where neither the start the nor the end activity type/location is home.

Home, work, and shopping are the predominant primary trip chain activities when people use automobile or NMT. For ride-hailing and transit trip chains, work and shopping are the most common primary activities. Additionally, the share of HBSOCREC trips, in the trip chain anchor activities category, is particularly high in ride-hailing trip chains compared to the other three modes.

Ride-hailing also has a noticeably high share—comparable to transit—in areas with higher residential density. High density or core areas of a city are usually served by a variety of transit systems as they can run efficiently in these high demand areas. Ride-hailing services also tend to operate efficiently in higher density areas as vehicles do not need to travel a long distance (or wait a long time) between dropping of a traveler and picking up the next traveler. This may indicate a potential substitution of ride-hailing for transit as ride-hailing services may provide a faster and more convenient travel option in some cases.

Table 2 also shows a statistically significant variation in the average household vehicle ownership in CBSA across the four modes. NMT and ride-hailing trip chain makers reside in areas with similar average household vehicle ownership. As expected, auto and transit trip chain makers live in areas with the highest and lowest average household vehicle ownership, respectively.

The descriptive statistics in Table 2 across the four modes provide a basis to estimate a trip chain choice model. The statistics indicate significant differences across the four modes in terms of who is choosing each mode, the structure and complexity of the trip chains, the primary activities associated with each mode, and even the residential density wherein the trips take place.

## Choice model results

This section presents and discusses the trip chain choice model estimation results. The Specification and Estimation of the Binary Logit Model subsection presents the final specification and parameter estimates for the BL model wherein the dependent variable denotes the existence of ride-hailing in a trip chain. The Specification and Estimation of the NL and MNL Model subsection presents the MNL and NL model specifications and parameter estimates. The dependent variable in both the MNL and NL models is the primary mode of the trip chain.

### Specification and estimation of the binary logit model

Table 4 displays the final specification, the parameter estimates, and the statistical significance of the parameter estimates and the odds ratio for the BL model. The magnitudes of the coefficients indicate the change in log odds of including ride-hailing in trip chain $i$ due to a unit change in the independent variable of interest. Positive parameter values indicate an increase in propensity to choose/use ride-hailing, in one or more trip segments, within a trip chain.

**Table 4:** Results of the BL model

Variables	Coefficient	z-Statistic	Odds Ratio
Intercept (Ride-hailing used in Trip Chain)	− 6.300***	− 7.790	0.002
Gender (Base = Male)			
Female	0.019	0.15	1.020
Age (Base = 16–35)			
36–65	− 1.059***	− 7.60	0.347
66 +	− 1.355***	− 4.68	0.258
Household income (Base = Low)			
Lower middle ($25,000 to < $50,000)	− 1.253***	− 3.850	0.286
Middle ($50,000 to < $100,000)	− 0.263	− 1.130	0.769
Upper middle ($100,000 to < $200,000)	− 0.176	− 0.740	0.839
High ($200,000 +)	0.637*	2.540	1.891
Ethnicity/race (Base = White)			
Black	0.112	0.420	1.118
Asian	− 0.071	− 0.340	0.932
Hispanic	− 0.109	− 0.500	0.897
Other race	− 0.255	− 0.680	0.775
Education (Base = Below Bachelor’s Degree)			
Above bachelor’s degree	0.389*	2.270	1.475
Life cycle status (Base = Working adult without child)			
Working adult with child 0–15	− 1.135***	− 5.410	0.321
Working adult with child 16–21	− 0.662*	− 2.160	0.516
Retired adult without children	− 0.512*	− 2.000	0.599
Employment status (Base = Unemployed)			
Part-time	− 0.394	− 1.520	0.675
Full-time	− 0.122	− 0.600	0.885
Public transit usage	0.023***	4.000	1.024
Vehicle availability (Base = < 1 vehicle per driver)			
High (1 + vehicle per driver)	− 0.650***	− 4.140	0.522
Travel day (Base = Weekend)			
Weekday	− 0.532***	− 3.450	0.587
Cumulative activity duration (mins)	0.005***	8.660	1.005
Cumulative travel distance (miles)	− 0.006*	− 1.990	0.994
Stops per trip chain	− 0.127*	− 2.220	0.881
Trip chain anchor activities (Base = HBW)			
HBSHOP	− 0.705	− 1.150	0.494
HBSOCREC	0.874**	2.980	2.396
HBO	− 0.770**	− 2.810	0.463
NHB	− 0.898***	− 5.190	0.407
Primary trip chain activity (Base = Home)			
Work	1.016***	3.400	2.762
School/daycare/religious	0.976*	2.510	2.653
Healthcare	1.515***	3.780	4.549
Shopping	1.201***	3.880	3.322
Social/recreational	1.628***	4.830	5.093
Drop-off/pickup	1.252***	3.540	3.497
Meal	0.882*	2.370	2.416
Other	0.963.	1.860	2.620
Residential density (Base = Low)			
Medium (500–1,999)	0.507	1.550	1.660
High (2,000–9,999)	1.499***	4.820	4.475
Very high (10,000–999,999)	2.764***	8.290	15.859
Average household vehicle ownership in CBSA	0.627.	1.860	1.872
Log−likelihood	− 1292.738		
LR χ2 or Wald χ2	962.620		
AIC	2665.477		
BIC	3017.794		

Note 1: All coefficient estimates are in reference to “no ride-hailing in trip chain”

Note 2: Sig. codes: '***' 0.001 '**' 0.01 '*' 0.05 '.' 0.10

The odds ratio represents the probability of ride-hailing existing in trip chain $i$ over the probability of ride-hailing not being in trip chain $i$, when an independent variable changes by one unit. Therefore, an odds ratio equal to 1, less than 1, or greater than 1 refers to a 50% probability, less than 50% probability and greater than 50% probability, respectively, of ride-hailing being in trip chain $i$, when there is one unit increase in the independent variable.

Most of the coefficient estimates in the statistical models in Table 4 are consistent with observations made in the Descriptive Analysis subsection from the descriptive statistics, even after controlling for potentially spurious correlations; however, several of the model parameters in Table 4 indicate statistically insignificant relationships.

Table 4 indicates that gender, ethnicity/race, and employment status do not have a statistically significant effect on the use of ride-hailing in trip chains. Conversely, persons aged 16–35, persons in households with annual incomes over $200,000, persons with a bachelor’s degree, workers without children, weekend travelers, persons who use public transit, persons in households with fewer than one vehicle per driver, persons who live in higher density residential areas and in areas with a higher number of vehicles per household have a positive and statistically significant relationship with ride-hailing usage in trip-chains. Nearly all these findings are consistent with the existing ride-hailing literature for non-trip-chaining, although the life cycle status (i.e., whether or not travelers have children) is not commonly incorporated in most existing models.

In terms of the magnitudes of the trip chain maker factors on use of ride-hailing, the parameter coefficients and the odds ratios indicate large impacts on ride-hailing usage in trip chains. The results indicate that the odds of someone over 65 years old including ride-hailing in their trip chain are 74% lower than someone between the ages of 16 and 35. Similarly, the odds of a working adult with a young child including ride-hailing in their trip chain are 68% lower than a working adult without children. Also, the odds of a person with more than one vehicle per household member in their house using ride-hailing in a trip chain are 48% lower than a person with more than one vehicle per household member. Moreover, compared with being from a low-income household and not having a bachelor’s degree, being from a high-income household and having a bachelor’s degree increases one’s odds of using ride-hailing in trip chains by 89% and 48% respectively.

The day of travel, weekend vs. weekday, also clearly plays a big role. Compared to a weekend day trip chain, the odds of a weekday trip chain including ride-hailing are 41% lower. This is a substantial difference and suggests ride-hailing plays a significantly bigger factor in trip chains occurring on weekends.

In addition to the attributes associated with trip chain makers and the areas in which they make trip chains, Table 4 includes attributes related to the complexity and structure of strip chain. According to Table 4, the inclusion of ride-hailing within a trip chain is also positively associated with total duration of activities in a trip chain, negatively correlated with the frequency of stops, and negatively correlated with cumulative travel distance. This set of results (total activity duration, stop frequency, total travel distance) indicate that travelers do not typically use ride-hailing in trip chains to complete many long-distance trips to activities; rather, travelers use ride-hailing in trip chains to travel relatively short distances between one or two relatively long duration secondary and/or primary activities. The odds ratio implies that increasing the stops per trip chain by one stop decreases the odds of using ride-hailing by 12%.

The trip chain anchor activities parameters in Table 4 suggest that trip chains forming between home and shopping, and two non-home activities are less likely to incorporate ride-hailing compared to trip chains forming between home and work; however, the difference is not statistically significant for home and shopping. Only HBSOCREC trip chains have a higher tendency to include ride-hailing than a HBW trip chain. These results suggest that ride-hailing currently plays the largest role in trip chains that include a social/recreational activity at one trip chain end and home as the other trip chain end, which is mostly unsurprising. The odds ratio for the trip chain anchor activities parameters indicates that the starting and ending anchor activities do play a big role in the inclusion of ride-hailing within trip chains.

Table 4 also shows a variety of results related to primary trip chain activity. All the non-home activities have a positive and statistically significant coefficient related to the home primary activity. The magnitudes for social/recreational and healthcare primary activities are noticeably large, indicating that all else being equal ride-hailing is quite frequently used for these two types of activities. The healthcare finding is particularly important, as it illustrates the possible important role ride-hailing plays in transporting travelers to healthcare activities along with intermediary activities along the way.

The residential density findings clearly illustrate their enormous impact on the use of ride-hailing within trip chains. The odds of trip chains within medium, high, and very high density areas including ride-hailing are 66%, 348%, and 1486% higher than trip chains in low density areas. Additionally, the average household vehicle ownership in the CBSA where the trip chain occurred implies that CBSA’s with higher vehicle ownership are significantly more likely to incorporate ride-hailing in trip chains.

The Discussion section explores the broader implications of several of these findings in more detail. Since, most of the socio-demographic and user travel characteristic results are consistent with the non-trip-chain ride-hailing literature, much of the discussion focuses on the results related to the trip chain structure, trip chain complexity, and activities associated with trip chains that include ride-hailing.

### Specification and estimation of the NL and MNL model

**Fig. 6:**
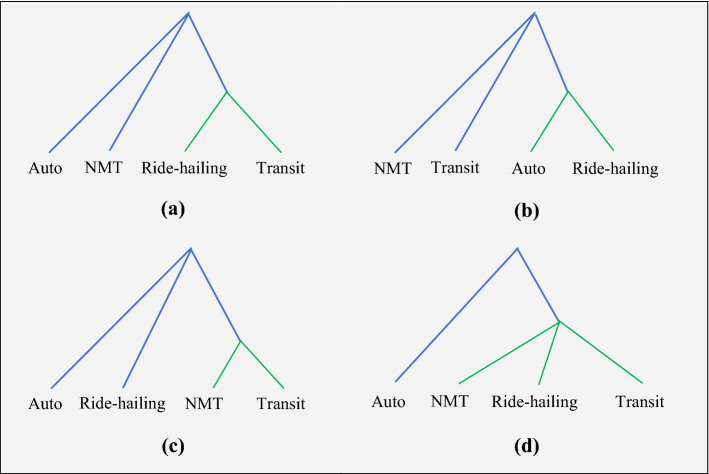
Alternative Nesting Structures for the Nested Logit Model

Figure 6 displays the four nesting structures considered and tested in this study. The logsum parameter ($\lambda$) for the degenerate nests (i.e., the nest with only one alternative) are constrained to unity. According to Ortúzar and Willumsen (2011), under this assumption, the nesting structure holds true if the estimated values of the logsum parameter fall in the range 0 < $\lambda$  < 1. This was found only for Nesting Structure (c), where NMT and transit share a nest, and ride-hailing and personal auto have their own degenerate nests. In Nesting Structure (c), the logsum parameter was significantly different than 1, with a value of 0.907. Hence, the final NL model structure is Nesting Structure (c).

Table 5 displays the results of the NL model wherein the dependent variable is the primary mode of the trip chain. The MNL model, with the same specification as the NL model, produced similar estimates and the results are provided in Table 7 in the Appendix. Both tables display the coefficient estimates of the model parameters, their statistical significance, and the odds ratio. For each mode alternative, the coefficients represent the change in log odds of choosing a mode over auto when there is a unit change in a particular independent variable. Similarly, the magnitudes of the odds ratio for each alternative mode indicates the probability of choosing that mode over the probability of choosing auto when a factor changes by one unit. This study includes four primary modes, namely, auto, NMT, ride-hailing, and transit, wherein auto is treated as the base alternative. Like the BL model, the NL and MNL models were specified considering the variables in Table 2 with the addition of average wait time.

**Table 5:** Results of the NL Model

Variables	Coefficients			z-statistic			Odds Ratio		
	NMT	Ride-hailing	Transit	NMT	Ride-hailing	Transit	NMT	Ride-hailing	Transit
Intercept	0.790*	− 6.416***	− 0.794.	2.350	− 5.190	− 1.720	2.203	0.002	0.452
Cumulative travel time (minutes)	− 0.031***			− 36.670			0.970		
Average transit wait time (minutes)	− 0.039***			− 3.430			0.962		
Gender (Base = Male)									
Female	− 0.199***	0.045	− 0.161.	− 3.370	0.240	− 1.940	0.820	1.046	0.852
Age (Base = 16–35)									
36–65	− 0.466***	− 1.214***	− 0.274**	− 6.650	− 5.770	− 2.840	0.627	0.297	0.760
66 +	− 0.897***	− 1.573***	− 0.627***	− 8.320	− 3.970	− 3.970	0.408	0.208	0.534
Household income (Base = Low)									
Lower middle ($25,000 to < $50,000)	− 0.080	− 0.858*	− 0.420**	− 0.760	− 1.990	− 2.760	0.923	0.424	0.657
Middle ($50,000 to < $100,000)	− 0.217*	− 0.080	− 0.491***	− 2.200	− 0.240	− 3.500	0.805	0.923	0.612
Upper middle ($100,000 to < $200,000)	− 0.115	− 0.050	− 0.246.	− 1.130	− 0.140	− 1.690	0.891	0.951	0.782
High ($200,000 +)	− 0.006	0.691.	− 0.275.	− 0.050	1.880	− 1.600	0.994	1.995	0.759
Ethnicity/race (Base = White)									
Black	− 0.252*	− 0.661	0.723***	− 2.000	− 1.440	5.280	0.777	0.516	2.060
Asian	0.207*	− 0.131	0.266.	2.080	− 0.440	1.920	1.230	0.877	1.305
Hispanic	− 0.157	− 0.738*	0.054	− 1.530	− 2.020	0.390	0.855	0.478	1.055
Other race	0.025	0.068	0.296	0.160	0.150	1.350	1.025	1.070	1.344
Education (Base = Below bachelor’s degree)									
Above bachelor’s degree	0.256***	0.645*	0.271**	3.780	2.460	2.670	1.291	1.906	1.311
Life cycle status (Base = Working adult without child)									
Working adult with child 0–15	− 0.027	− 1.153***	− 0.457***	− 0.350	− 3.540	− 4.020	0.973	0.316	0.633
Working adult with child 16–21	− 0.379**	− 1.027*	− 0.187	− 2.870	− 2.050	− 1.080	0.685	0.358	0.829
Retired adult without children	− 0.239*	− 0.535	− 0.323*	− 2.460	− 1.490	− 2.310	0.787	0.586	0.724
Employment status (Base = Unemployed)									
Part-time	− 0.193.	− 0.868*	− 0.755***	− 2.000	− 2.330	37.160	0.825	0.420	0.470
Full-time	− 0.545***	− 0.818**	− 0.881***	− 6.560	− 2.860		0.580	0.441	0.414
Public transit usage	0.084***	0.105***	0.201***	15.550	9.480	37.160	1.088	1.111	1.223
Vehicle availability (Base = < 1 vehicle per driver)									
High (1 + vehicle per driver)	− 0.117	− 0.340	− 0.275**	− 1.500	− 1.550	− 2.750	0.890	0.712	0.759
Travel day (Base = Weekend)									
Weekday	0.085	− 0.331	1.219***	1.180	− 1.430	8.990	1.088	0.718	3.385
Cumulative activity duration	0.001***	0.006***	0.004***	3.860	6.510	10.130	1.001	1.006	1.004
Stops per trip chain	− 0.302***	− 0.455***	− 0.270***	− 9.530	− 4.800	− 6.820	0.739	0.634	0.763
Trip chain anchor activities (Base = HBW)									
HBSHOP	− 0.237	− 0.267	− 2.092***	− 1.320	− 0.410	− 5.130	0.789	0.766	0.123
HBSOCREC	0.404.	1.567***	− 0.548	1.700	4.190	− 1.630	1.497	4.792	0.578
HBO	− 0.450***	− 0.806*	− 1.367***	− 3.460	− 2.270	− 8.260	0.637	0.447	0.255
NHB	0.359***	− 1.080***	− 1.093***	3.410	− 4.030	− 8.650	1.433	0.339	0.335
Primary trip chain activity (Base = Home)									
Work	0.238**	1.462***	0.650***	2.750	3.500	4.370	1.269	4.313	1.915
School/daycare/religious	− 0.295*	1.253*	0.232	− 2.130	2.280	1.060	0.745	3.500	1.260
Healthcare	− 0.089	1.943***	0.852***	− 0.480	3.480	3.600	0.915	6.982	2.345
Shopping	− 0.431***	1.299**	0.204	− 4.290	2.950	1.250	0.650	3.666	1.226
Social/Recreational	0.075	1.879***	0.686***	0.550	3.970	3.300	1.078	6.549	1.985
Drop−off/pickup	− 0.474***	1.388**	0.534**	− 3.290	2.640	2.790	0.622	4.005	1.706
Meal	− 0.177	1.362**	0.473*	− 1.330	2.660	2.330	0.837	3.902	1.605
Other	0.094	1.457*	0.688*	0.480	2.000	2.390	1.099	4.294	1.989
Residential density (Base = Low)									
Medium (500–1,999)	0.003	0.857	0.339.	0.030	1.370	1.930	1.003	2.355	1.404
High (2,000–9,999)	0.587***	1.684**	0.876***	5.790	2.790	5.190	1.799	5.387	2.402
Very high (10,000–999,999)	1.671***	3.493***	1.562***	12.680	5.660	8.080	5.320	32.868	4.767
Average household vehicle ownership in CBSA	− 0.341*	0.678	− 1.415***	− 2.380	1.400	− 7.360	0.711	1.970	0.243
Log-likelihood	− 6949.174								
LR χ2 or Wald χ2	3941.550 (*p* = 0.000)								
AIC	14,138.350								
BIC	15,361.070								
Dissimilarity parameter ($$\uplambda$$)	0.907								
LR test for IIA ($$\uplambda$$ = 1): χ2	4.370 (*p* = 0.037)								

Note 1: N = 46,115, 1,933, 141 and 1,222 for Auto, NMT, Ride-hailing and Transit, respectively

Note 2: All coefficient estimates are in reference to the choice of trip chains with Auto

Note 3: Sig. codes: '***' 0.001 '**' 0.01 '*' 0.05 '.' 0.10

The NL model includes a statistically significant logsum parameter of 0.907 for the nest containing NMT and transit, suggesting a correlation between the error components of these two alternatives. In terms of model fit, the NL model is similar to the MNL model across all relevant metrics including log-likelihood, AIC, and BIC. Given the statistical significance of the NL logsum parameter, the following discussion will focus on the NL model results.

#### Alternative-specific variables

Among the explanatory variables, only cumulative trip chain travel time and average transit wait time were specified as alternative-specific variables. Please see the Descriptive Analysis subsection for a description of how cumulative travel time is calculated for each mode. The coefficients for the alternative-specific variables in both models indicate, consistent with basic transportation theory, that the propensity of choosing a trip chain mode decreases with a rise in the cumulative trip chain travel time and average transit wait time. Based on these two parameter values, the disutility for average wait time is higher than cumulative travel time but the ratio of the two is smaller than much of the existing literature (Frank et al. 2008; Frei et al. 2017; Idris et al. 2015; Wardman 2004). The odds ratios imply that a one-minute increase in travel time in mode $m$ and a one minute increase in transit wait time $m$, reduce the odds of a trip chain maker choosing mode $m$ by approximately 3% and 4%, respectively.

#### Choice of ride-hailing versus auto

According to Table 5, considering only the statistically significant parameters, ride-hailing services for trip chaining are preferred by people who are younger (16–35 years), are from high-income households, are non-Hispanic, have high educational attainment, and are working adults without children. Additionally, in the case where unemployed is the base for employment status, both part-time and full-time coefficients are negative, statistically significant, and nearly equal in magnitude. This indicates that workers are less inclined to use ride-hailing in trip chains irrespective of their work hour duration, compared to unemployed persons. The odds ratios for trip chain makers older than 65, who are working with a child, and who work part time suggest compared to trip chain makers between 16 and 35, who are working without children, and who do not work, indicate these factors significantly decrease the propensity to choose ride-hailing as the primary trip chain mode compared to auto. The converse is true for high-income trip chain makers and trip chain makers with a bachelor’s degree who are much more likely, to choose ride hailing as their primary trip chain mode compared to the low-income trip chain maker and trip chain makers without a bachelor’s degree.

Interestingly, the weekend parameter is statistically insignificant in the case of primary trip chain mode for ride-hailing. This suggests that while Table 4 shows that the day of the week plays a big role for the existence of ride-hailing in trip chains, Table 5 indicates it does not play a significant role in the choice of ride-hailing as the primary trip chain mode. This requires further investigation, but one possibility is that users of ride-hailing within trip chains are fundamentally different than trip chain makers who use ride-hailing as their primary mode, and the former group may have a series of activities that are amenable to trip chaining with ride-hailing on the weekend but not weekdays.

Ride-hailing trip chain makers are also found to be more frequent transit users, which is similar to the BL model results. However, although vehicle availability (represented by vehicle per driver) has a negative parameter value, the parameter is insignificant in the NL model for ride-hailing. Also, unlike the BL model, the coefficient representing average household ownership in the home CBSA of the traveler is insignificant.

The cumulative activity duration and stops per trip chain parameter values are similar in the NL and BL models for ride-hailing, with ride-hailing positively correlated with cumulative activity duration and negatively correlated with stops per trip chain. This once again indicates that trip chain makers tend to use ride-hailing to travel between a few activities with long durations. However, while the odds ratios are not directly comparable between the BL and NL model, the odds ratio, as hypothesized, is significantly lower in the NL model for the stops per trip chain factor than the BL model.

The primary trip chain activity results indicate that travelers are least likely to have home as the primary activity associated with a trip chain. Among the non-home primary activities, healthcare and social/recreational activities are the most likely to be associated with a ride-hailing trip chain. The significantly positive coefficient of HBSOCREC for trip chain anchor activities category with the base being HBW also supports this finding.

The results also indicate a very strong relationship between the residential density of the trip chain’s location and ride-hailing as the primary trip chain mode. As residential density increases, the model results indicate a statistically and steady (in terms of magnitude) increase in the propensity of trip chain makers to choose ride-hailing. As residential density goes from low to high and low to very high, the odds of a trip chain maker choosing ride-hailing over choosing auto increase by 439% and 3187%, respectively. These increases are substantially higher for ride-hailing than even transit and NMT, despite these latter two modes being associated with high usage in dense urban areas.

#### Similarities and differences between NMT, transit and ride-hailing in trip chains

An in-depth discussion of the NMT and transit parameters is beyond the scope of this study. However, the differences between NMT and transit and ride-hailing parameters are worth noting. According to the model results, while gender is insignificant for ride-hailing and transit, it is a significant factor for NMT with a negative coefficient for female. Additionally, in terms of race, Black trip chain makers have a positive coefficient for transit, negative for NMT, and negative but statistically insignificant for ride-hailing. Also, while the vehicle availability parameter is statistically significant and negative for transit, it is insignificant for NMT and ride-hailing. Transit has significant and positive coefficient for weekday trip chains, which indicates a greater tendency of trip chain makers to incorporate this mode in trip chains formed on weekdays compared to weekends. This contrasts with the negative but statistically insignificant coefficient for weekday trip chains for ride-hailing and the positive but statistically insignificant coefficient for NMT.

Regarding the trip chain anchor activities variable, the coefficients suggest transit is primarily used for HBW tours, whereas ride-hailing is primarily used for HBSOCREC tours, and NMT for NHB tours. The parameters for the three non-auto modes are distinct in the trip chain anchor activities category. If planners or policymakers are looking to plan a transportation system that reduces dependency on private auto, the differences in the trip chain activity parameters for NMT, transit, and ride-hailing suggest that each different mode serves different, complementary, purposes and they may all be needed to allow travelers to forego auto ownership or near exclusive auto usage. Discussion.

## Discussion

### Consistency of results with study hypotheses

The results from the BL, MNL and NL models support the high-level hypotheses laid out in the Research Hypotheses subsection that modal attributes, trip chain maker characteristics, activity types, trip chain complexity and structure, and land-use characteristics impact the propensity of trip chain makers to use ride-hailing with trip chains. The following three subsection focus on three particularly important and interesting relationships between explanatory variables and ride-hailing usage.

#### Ride-hailing and primary trip chain activity

The choice of ride-hailing within trip chains is strongly associated with healthcare as a primary activity which signifies the important role ride-hailing services currently play in providing access to healthcare facilities. Considering the negative association between ride-hailing use and vehicle availability in the BL model, it is also plausible that people with zero or low access to personal vehicles use ride-hailing services as a substitute (in at least one part of trip chain) to access essential services, where healthcare is just one example.

The healthcare finding is arguably the most important in the study. It indicates that ride-hailing plays a critical role in the current transportation system. Policymakers interested in ensuring access to healthcare and other essential services across ages, incomes, races, and genders, may consider ensuring access to the ride-hailing services that can and currently do provide transport to healthcare facilities. This may take the form of subsidies for riders to make certain trips. Or it may take the form of working with health insurers and healthcare providers to promote ride-hailing as a means of transport to healthcare facilities.

The particularly interesting thing about this finding is that even when travelers chain multiple trips on the way to the primary healthcare activity, ride-hailing still plays an important role. This means that travelers are not just ride-hailing from home to a healthcare facility. They are conducting other secondary activities before arriving at a healthcare facility. This further indicates the potential value of ride-hailing as a mode that enables trip chaining.

The choice of ride-hailing within trip chains is also strongly associated with social/recreational primary activities. Although this is an unsurprising finding, it is nevertheless important to understand the role ride-hailing plays in the current transportation system. The model results in this paper indicate that ride-hailing does play an important role connecting travelers to social/recreational activities, even in the context of trip chaining.

#### Ride-hailing and trip chain attributes

The model contains two main attributes to capture trip chain structure and complexity, namely, activity duration and stops per trip chain. More stops per trip chain coincide with a more complex trip chain, as they require more cognitive effort to sequence the activities, schedule the activities and trip start times, and determine modes and routes to travel between activity pairs. Although maybe less obvious, longer activity durations are also assumed to increase complexity because, all else being equal, longer activities by definition consume more hours in a day. A reduction in available hours per day to move between activity locations effectively constrains a trip chain maker’s ability to sequence and schedule travel between activities as well as execute travel itself.

The findings in Table 5 indicate that longer activity durations and fewer stops per trip chain increase the likelihood of users choosing NMT, ride-hailing, and transit relative to the personal vehicle. However, the magnitude is highest for ride-hailing across both attributes. Looking at stops per trip chain, the results indicate that the private auto dominates trip chains with more stops (i.e., higher complexity or level of difficulty). Looking at activity duration, it appears that when activity durations increase, non-auto modes are more prevalent. Hence, it appears that auto is most valuable when a trip chain maker needs to make numerous stops, where the cumulative stop time is relatively short.

In previous research, Ye et al. (2007) examine the impact of trip chain complexity on mode choice in work and non-work tours. Following some of the suggestions (pertaining to transit) provided in Ye et al. (2007), an increase in ridership in ride-hailing and/or other shared mobility services (e.g., conventional ridesharing, ride-splitting, bikesharing, micro-transit, etc.) could be achieved by locating multiple and diverse activities at a single location in order to allow trip chain makers to satisfy their needs/demand for multiple activity types while reducing the number of stops.

#### Ride-hailing and trip chain maker attributes

Results from BL and NL models also reveal some important findings relevant to trip chain makers and their propensity to use ride-hailing. While some of the variables like age, household income, and auto ownership have their usual association with the use of ride-hailing (independent of trip chains) reported in recent studies (Alemi et al. 2018; Feigon and Murphy 2016), there are additional factors that appear to be influenced by trip chaining. For example, ride-hailing trip chains are less popular among workers who have children aged 0–15 years. These parents would likely be highly inconvenienced by the lack of a car seat in ride-hailing (and/or transit) vehicles and would likely rely on their own personal auto to complete trip chains. This also implies that special care may need to be taken for parents of young children within ride-hailing services if ride-hailing companies want to serve this market.

Another notable modeling result is that frequent transit users have a high likelihood of using ride-hailing (and transit) for trip chains. This suggests that ride-hailing services are providing significant mobility benefits, in the context of trip chains, to public transit users.

The model results also indicate that residential density has a very strong association with ride-hailing trip chaining. As discussed by Conway et al. (2018), the relationship is reasonable as ride-hailing trips are usually shorter and less costly in dense areas, where activities are closer and parking is very expensive and/or very difficult to find. A particularly striking finding is that the effect of density on ride-hailing is at least twice the size of density’s effect on NMT and transit, despite NMT and transit usage being known to have a strong positive relationship with density (Chakrabarti and Shin 2017; Saelens and Handy 2008). With trip chaining, the higher preference for ride-hailing services in high density areas could result from the low wait times and the need to make fewer stops (due to activity clustering) in dense areas. The conjecture regarding activity clustering in dense areas is supported by the relatively high walk trip percentage as a secondary mode associated with ride-hailing found in Fig. 3.

### Consistency of results with existing literature

In addition to the novel findings discussed in the previous section, there are also some findings which are inconsistent with the existing trip chain literature. Researchers analyzing trip chaining behavior have observed that women are usually more involved in trip chains than men, particularly in households with children (Kumar and Levinson 1995; McGuckin and Murakami 1999). However, this study did not find any significant difference between the trip chain mode preference of women and men for ride-hailing. The results only shows that women have a lower tendency to choose NMT and transit than men. This outcome could be due to the current models’ inability to distinguish mode preference across different trip purposes and gender. Moreover, the trip chain mode choice may be significantly influenced by the number and age group of children on the trip, which the models do not incorporate due to data availability.

## Conclusion

### Summary

The personal auto offers many advantages over transit and NMT modes in terms of chaining trips. Vehicle-based mobility services such as ride-hailing offer many of the trip chain advantages of a personal auto including scheduling and route flexibility compared to transit and short travel times compared NMT modes. However, with ride-hailing services, travelers face difficulty when traveling requires moving with additional items, like a child car seat or even groceries and other shopping items. Moreover, ride-hailing is a relatively expensive travel mode. Given the similarities and differences between ride-hailing and the personal auto in terms of completing trip chains, this study aims to assess the attractiveness of ride-hailing as a trip-chain mode. To this end, this study estimates a BL model, an MNL model, and a NL model to explicate the choice of ride-hailing as a mode in any segment of the trip chain and also as a primary trip chain mode (based on distance).

The modeling results include the novel findings that ride-hailing trip chains are more likely to terminate in healthcare and social/recreational activities than auto, NMT, and transit. The social/recreational findings are unsurprising given the clear benefit of not needing to drive to/from events where alcohol may be consumed and/or parking may be expensive. The healthcare finding is particularly interesting, as it indicates ride-hailing provides travelers who need healthcare a valuable travel option. Moreover, the significantly high coefficient for healthcare in the case of ride-hailing suggests a potential role for planners and policymakers or even healthcare providers in leveraging ride-hailing to further improve access to healthcare facilities. Making healthcare facilities accessible via ride-hailing may entail designating pickup and drop-off locations at healthcare facilities for ride-hailing vehicles or incentivizing ride-hailing companies to transport travelers to healthcare facilities that are located near the suburban-rural or suburban-exurban divide.

Several findings in this study are consistent with observations in previous ride-hailing studies that focus on individual trips rather than trip chains (Alemi et al. 2018; Dias et al. 2017; Feigon and Murphy 2016). For example, this study and previous studies find positive relationships between ride-hailing and persons who: are younger, highly educated, live in high-income households, use public transit frequently, and reside in high-density areas. In addition, there are similar findings on increased tendency to use ride-hailing during weekends.

Another takeaway from this study is that persons who use ride-hailing for trip chaining are also frequent transit riders. While a single cross-section of trip chaining trips does not permit strong claims about whether ride-hailing is a complement or substitute to transit, the empirical finding in this study clearly indicates a relationship between the two modes in the context of trip chaining. In the case where ride-hailing does complement transit on an individual trip level, there is a need to plan and manage a multi-modal transportation system to integrate public transit and ride-hailing services. However, in the NHTS data, the percentage of ride-hailing trips that act as first- or last-mile feeder to transit within trip chains is less than 10% of all ride-hailing trips, indicating that ride-hailing is not a substantial complement to transit in this way. Nevertheless, there are other mechanisms by which ride-hailing and transit may be complementary services within trip chaining, namely, ride-hailing may allow travelers to forego vehicle ownership or purchasing an additional vehicle, thereby resulting in travelers substituting both ride-hailing and transit trips for previous personal auto trips. Similarly, ride-hailing may complement transit via enabling travelers to take transit to a major activity center during the peak period when the traveler also needs to travel to areas that are not well connected with transit during the off-peak period but can be served by ride-hailing.

The study also indicates that as trip chain complexity increases, ride-hailing tends to be the least preferred primary trip chain mode. Trip chain makers are less likely to use ride-hailing with an increase in trip chain stops (compared to personal automobile) than even transit and NMT. This finding suggests that the benefits of a personal auto for trip chaining, relative to a ride-hailing service for trip chaining, are quite significant and may limit the ability of households to forego auto ownership.

### Limitations

To separate ride-hailing users from the NHTS combined category for ride-hailing/taxi, this study uses the data on TNC app usage and assumes that people who used the ride-hailing app at least once in the past 30 days are ride-hailing users. Although this is a strong assumption, this is the only information available which could reasonably differentiate ride-hailing users from taxi users. The authors anticipate and hope that future NHTS data will distinguish between ride-hailing and taxi trips. The dataset is also missing relevant modal attributes that would be useful for a mode choice analysis, such as travel cost, wait time, transit transfers, etc. The analysis would also benefit from a higher spatial resolution (e.g., census tracts or block groups), trip destination location information, and a distinction between delivery work trips (i.e., food delivery) and other work trips.

The study is also limited by several assumptions that support the analyses. For example, it is assumed that mode choice is dependent on trip chain complexity, not the other way around. Although this assumption is supported by Ye et al. (2007) using Swiss Travel Survey dataset, the order of preference between choice of mode and trip chain complexity has not been investigated using the 2017 NHTS dataset.

This study also does not consider the trip making dependency between household members that can influence trip chaining characteristics. In households, some trips or activities can be shared (e.g., recreation, eat-out, etc.), whereas others are carried out by one of the household members (e.g., grocery shopping, dropping-off or picking-up children from school). In case of the latter, it is highly likely that with the increase in one household member’s trip chaining complexity, there will be a decrease in the trip chaining complexity of the other household member. An investigation of this interrelationship between the trip chaining pattern of household members would require the incorporation of household activity distribution into the modeling framework, which we aim to explore in future extensions of this study.

In terms of the model structure, the study assumes a causal, one-directional, relationship between each of the explanatory variables and either the choice to include ride-hailing within a trip chain or the choice of ride-hailing as the primary trip chain mode. A more complex model structure, such as structural equation models, can test and capture for simultaneity and/or reverse causation. Examples of potential simultaneity or reverse causality include public transit usage, vehicle availability, cumulative activity duration, and stops per trip chain. The usage of ride-hailing in general as well as within trip chains may cause transit usage to increase directly or indirectly through decreases in car ownership. As such, it is also conceivable that ride-hailing propensity (within trip chains) impacts car ownership. Finally, if mode choice and trip chain structure decisions are made jointly, then simultaneity bias may impact the coefficient estimates for number of stops—a personal auto enables more stops than NMT, transit, or ride-hailing in most cases—and cumulative activity duration—as slower modes reduce the amount of time travelers have to conduct activities.

### Future research

Although this study provides valuable insights into the role of ride-hailing within trip chains, the data limitations mentioned in the previous subsection limit the range of research questions related to ride-hailing and trip chains that can be answered. Hence, an important future research direction involves collecting different types of data on the propensity of travelers to use ride-hailing within trip chains. Both panel surveys that capture behavioral changes over time and stated preference surveys that allow hypothetical trip chaining options could provide deeper insights into the role of ride-hailing within trip chains.

Specifically, panel and stated preference surveys could provide insights into the modal substitution effects between ride-hailing and transit within trip chains and between ride-hailing and personal auto within trip chains. Understanding these substitution effects is critical to developing policies to make urban transportation systems more sustainable and efficient.

Finally, the relationship between ride-hailing trip chains and residential density found in this study necessitates further exploration because the demand for many auto trips within a short period in a core area can worsen congestion, if ride-hailing replaces walking, biking, or transit and if ride-hailing services continue to have high deadheading miles between occupied travel. This issue is important because up to 40% of the ride-hailing trips are observed to operate in peak hours in several high-density urban areas like Los Angeles, San Francisco, Chicago, New York, and Boston (Feigon and Murphy 2018; Gehrke et al. 2018). In general, with the availability of more detailed data on the location of activities and individual trips (e.g., number of items carried by the trip makers, scheduling or routing constraints, etc.), the authors would like to extend the research to include more built environment factors and compare the trip chaining pattern of ride-hailing trips with unchained trips.

## Figures and Tables

**Table 6:**
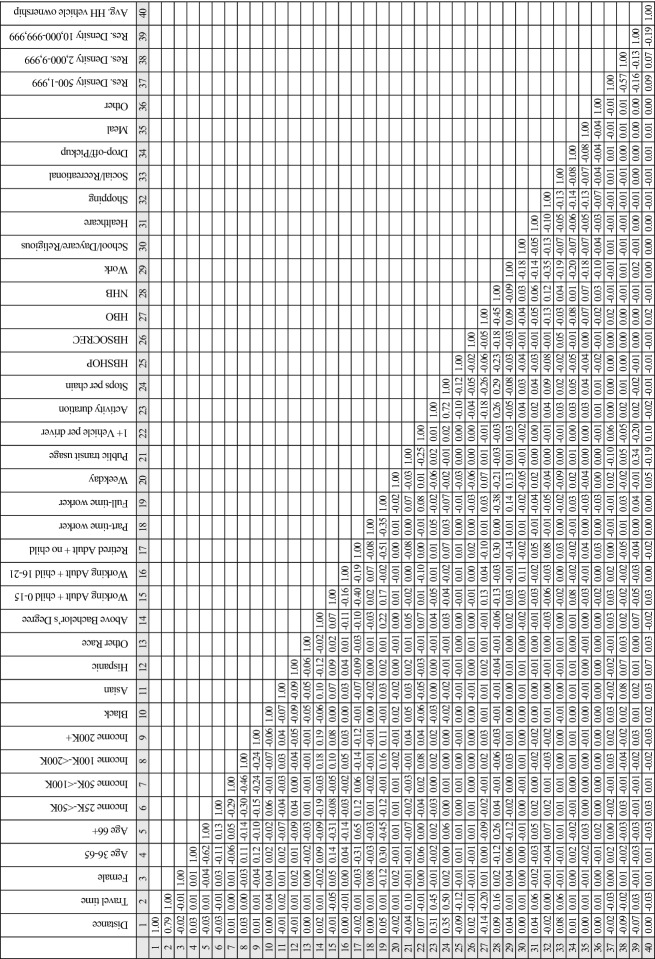
Correlation matrix of the model variables

## Data Availability

All the analyses conducted in this study relied exclusively on the publicly available 2017 National Household Travel Survey dataset.

## Appendix

See Tables 6 and

**Table 7:** Results of the MNL model

Variables	Coefficients			z-Statistic			Odds Ratio		
	NMT	Ride-hailing	Transit	NMT	Ride-hailing	Transit	NMT	Ride-hailing	Transit
Intercept	0.743*	− 6.424***	− 0.841.	2.180	− 5.180	− 1.760	2.102	0.002	0.431
Cumulative travel time (minutes)	− 0.031***			− 38.700			0.969		
Average transit wait time (minutes)	− 0.039***			− 3.210			0.962		
Gender (Base = Male)									
Female	− 0.199***	0.047	− 0.167.	− 3.340	0.260	− 1.940	0.820	1.048	0.847
Age (Base = 16–35)									
36–65	− 0.471***	− 1.221***	− 0.265**	− 6.650	− 5.800	− 2.650	0.624	0.295	0.767
66 +	− 0.902***	− 1.583***	− 0.624***	− 8.280	− 3.990	− 3.800	0.406	0.205	0.536
Household income (Base = Low)									
Lower middle ($25,000 to < $50,000)	− 0.080	− 0.857*	− 0.426**	− 0.750	− 1.980	− 2.690	0.923	0.424	0.653
Middle ($50,000 to < $100,000)	− 0.217*	− 0.072	− 0.495***	− 2.170	− 0.220	− 3.380	0.805	0.930	0.610
Upper middle ($100,000 to < $200,000)	− 0.117	− 0.039	− 0.239	− 1.140	− 0.110	− 1.580	0.889	0.962	0.787
High ($200,000 +)	− 0.007	0.702.	− 0.282	− 0.060	1.910	− 1.570	0.993	2.017	0.754
Ethnicity/race (Base = White)									
Black	− 0.269*	− 0.660	0.778***	− 2.100	− 1.440	5.630	0.764	0.517	2.177
Asian	0.208*	− 0.137	0.272.	2.070	− 0.460	1.890	1.231	0.872	1.313
Hispanic	− 0.159	− 0.747*	0.066	− 1.530	− 2.030	0.460	0.853	0.474	1.069
Other race	0.027	0.057	0.291	0.180	0.130	1.270	1.028	1.059	1.338
Education (Base = Below bachelor’s degree)									
Above bachelor’s degree	0.252***	0.652*	0.282**	3.700	2.480	2.680	1.287	1.919	1.325
Life cycle status (Base = Working adult without child)									
Working adult with child 0–15	− 0.022	− 1.156***	− 0.484***	− 0.280	− 3.550	− 4.120	0.978	0.315	0.616
Working adult with child 16–21	− 0.388**	− 1.035*	− 0.171	− 2.910	− 2.060	− 0.960	0.678	0.355	0.843
Retired adult without children	− 0.240*	− 0.533	− 0.321*	− 2.450	− 1.480	− 2.210	0.787	0.587	0.725
Employment status (Base = Unemployed)									
Part-time	− 0.190.	− 0.874*	− 0.770***	− 1.960	− 2.340	− 4.740	0.827	0.417	0.463
Full-time	− 0.542***	− 0.823**	− 0.899***	− 6.460	− 2.870	− 6.500	0.581	0.439	0.407
Travel day (Base = Weekend)									
Weekday	0.068	− 0.325	1.303***	0.940	− 1.400	9.520	1.070	0.722	3.681
Public transit usage	0.081***	0.106***	0.206***	15.070	9.440	41.800	1.085	1.112	1.229
Vehicle availability (Base = < 1 vehicle per driver)									
High (1 + vehicle per driver)	− 0.124	− 0.335	− 0.263**	− 1.580	− 1.520	− 2.540	0.884	0.715	0.768
Cumulative activity duration	0.001***	0.006***	0.004***	3.730	6.520	10.270	1.001	1.006	1.004
Stops per trip chain	− 0.303***	− 0.454***	− 0.262***	− 9.430	− 4.780	− 6.410	0.738	0.635	0.770
Trip chain anchor activities (Base = HBW)									
HBSHOP	− 0.200	− 0.273	− 2.206***	− 1.110	− 0.420	− 5.170	0.819	0.761	0.110
HBSOCREC	0.450.	1.562***	− 0.620.	1.880	4.170	− 1.770	1.568	4.767	0.538
HBO	− 0.421***	− 0.816*	− 1.428***	− 3.190	− 2.300	− 8.430	0.656	0.442	0.240
NHB	0.393***	− 1.097***	− 1.183***	3.680	− 4.090	− 9.580	1.482	0.334	0.306
Primary trip chain activity (Base = Home)									
Work	0.248**	1.468***	0.657***	2.840	3.510	4.240	1.281	4.340	1.930
School/daycare/religious	− 0.294*	1.263*	0.244	− 2.100	2.300	1.070	0.746	3.535	1.276
Healthcare	− 0.077	1.951***	0.867***	− 0.410	3.490	3.520	0.926	7.033	2.381
Shopping	− 0.427***	1.300**	0.207	− 4.210	2.950	1.220	0.652	3.669	1.230
Social/recreational	0.078	1.884***	0.708***	0.570	3.970	3.290	1.081	6.583	2.031
Drop−off/pickup	− 0.491***	1.399**	0.574**	− 3.350	2.660	2.900	0.612	4.053	1.775
Meal	− 0.178	1.365**	0.494*	− 1.320	2.660	2.340	0.837	3.916	1.639
Other	0.099	1.465*	0.695*	0.510	2.010	2.320	1.104	4.328	2.004
Residential density (Base = Low)									
Medium (500–1,999)	0.0002	0.855	0.338.	0.000	1.370	1.870	1.000	2.351	1.402
High (2,000–9,999)	0.590***	1.681**	0.852***	5.770	2.780	4.900	1.805	5.370	2.345
Very high (10,000–999,999)	1.691***	3.492***	1.508***	12.710	5.660	7.620	5.425	32.847	4.519
Average household vehicle ownership in CBSA	− 0.311*	0.675	− 1.504***	− 2.150	1.390	− 7.730	0.733	1.964	0.222
Log-likelihood	− 6951.360								
LR χ2 or Wald χ2	4957.260 (*p* = 0.000)								
AIC	14,140.720								
BIC	15,353.250								

Note 1: N = 46,115, 1,933, 141 and 1,222 for Auto, NMT, Ride-hailing and Transit, respectively

Note 2: All coefficient estimates are in reference to the choice of trip chains with Auto

Note 3: Sig. codes: '***' 0.001 '**' 0.01 '*' 0.05 '.' 0.10

^7^.

## Code availability

The authors used Stata (Version 13.0) for data preparation, descriptive statistics, and model estimation. Both built-in and community-contributed packages of Stata were employed.
